# c-kit Haploinsufficiency impairs adult cardiac stem cell growth, myogenicity and myocardial regeneration

**DOI:** 10.1038/s41419-019-1655-5

**Published:** 2019-06-04

**Authors:** Iolanda Aquila, Eleonora Cianflone, Mariangela Scalise, Fabiola Marino, Teresa Mancuso, Andrea Filardo, Andrew J. Smith, Donato Cappetta, Antonella De Angelis, Konrad Urbanek, Andrea M. Isidori, Michele Torella, Valter Agosti, Giuseppe Viglietto, Bernardo Nadal-Ginard, Georgina M. Ellison-Hughes, Daniele Torella

**Affiliations:** 1 Molecular and Cellular Cardiology, Department of Experimental and Clinical Medicine, Magna Graecia University, Catanzaro, 88100 Italy; 2 Faculty of Biological Sciences, School of Biomedical Sciences, University of Leeds, Leeds, LS2 9JT UK; 3 Section of Pharmacology, Department of Experimental Medicine, University of Campania “L.Vanvitelli”, Naples, 80121 Italy; 4 Department of Experimental Medicine, “La Sapienza” University, Rome, 00161 Italy; 5 Department of Cardiothoracic Surgery, University of Campania “L.Vanvitelli”, Naples, 80121 Italy; 6 Interdepartmental Center of Services (CIS) and Department of Experimental and Clinical Medicine, University Magna Graecia, Campus Salvatore Venuta-Viale Europa, Catanzaro, 88100 Italy; 7 Faculty of Life Sciences, School of Basic and Medical Biosciences, King’s College London, Guy’s Campus, London, SE1 1UL UK

## Abstract

An overdose of Isoproterenol (ISO) causes acute cardiomyocyte (CM) dropout and activates the resident cardiac c-kit^pos^ stem/progenitor cells (CSCs) generating a burst of new CM formation that replaces those lost to ISO. Recently, unsuccessful attempts to reproduce these findings using c-kit^Cre^ knock-in (KI) mouse models were reported. We tested whether c-kit haploinsufficiency in c-kit^Cre^KI mice was the cause of the discrepant results in response to ISO. Male C57BL/6J wild-type (wt) mice and c-kit^Cre^KI mice were given a single dose of ISO (200 and/or 400 mg/Kg s.c.). CM formation was measured with different doses and duration of BrdU or EdU. We compared the myogenic and regenerative potential of the c-kit^Cre^CSCs with wtCSCs. Acute ISO overdose causes LV dysfunction with dose-dependent CM death by necrosis and apoptosis, whose intensity follows a basal-apical and epicardium to sub-endocardium gradient, with the most severe damage confined to the apical sub-endocardium. The damage triggers significant new CM formation mainly in the apical sub-endocardial layer. c-kit haploinsufficiency caused by c-kit^Cre^KIs severely affects CSCs myogenic potential. c-kit^Cre^KI mice post-ISO fail to respond with CSC activation and show reduced CM formation and suffer chronic cardiac dysfunction. Transplantation of wtCSCs rescued the defective regenerative cardiac phenotype of c-kit^Cre^KI mice. Furthermore, BAC-mediated transgenesis of a single c-kit gene copy normalized the functional diploid c-kit content of c-kit^Cre^KI CSCs and fully restored their regenerative competence. Overall, these data show that c-kit haploinsufficiency impairs the endogenous cardioregenerative response after injury affecting CSC activation and CM replacement. Repopulation of c-kit haploinsufficient myocardial tissue with wtCSCs as well c-kit gene deficit correction of haploinsufficient CSCs restores CM replacement and functional cardiac repair. Thus, adult neo-cardiomyogenesis depends on and requires a diploid level of c-kit.

## Introduction

The lack of curative therapies for heart failure (HF)^1^ has made the search for an effective cellular replacement of lost/damaged cardiomyocytes (CMs) a priority in the war against HF epidemics^1,2^. Ex-vivo expanded resident cardiac stem cells (CSCs) and progenitor cells have been reproducibly shown to repair extensive myocardial damage and regenerate lost CMs^3–6^. Nevertheless, it is argued^7–9^ that the myocardium lacks a bona fide endogenous CM-generating progenitor cell population of biological significance.

This controversy has been mainly nurtured by Cre-lox mouse lines with Cre knocked-in (KI) to the c-kit locus (c-kit^Cre^KI)^10–13^. c-kit^Cre^KI lines show that myocardial c-kit-expressing (c-kit^pos^) cells only minimally, if not negligibly, contribute CMs^10–14^. However, the very low number of endogenous c-kit^pos^CSC-generated CMs detected in the c-kit^Cre^ mice, simply reflects the failure of the c-kit^Cre^-null allele produced by Cre insertion to recombine the CSCs and to track their progeny together with the severe defect in CSCs myogenesis produced by the c-kit hemizygosity^15–18^.

Recently, the deleterious effects of excessive catecholamines have been linked to the development of Takotsubo syndrome, a stress-related form of acute severe, generally transient, left ventricular (LV) dysfunction^19^. In rats and mice, a single excessive dose injection (subcutaneous (s.c.)) of the synthetic catecholamine, isoproterenol (ISO), causes CM death^20,21^, which leads to the development of acute HF, fully reversible by 28 days. Using this acute ISO-damage model, by means of several genetic tests and selective cell ablation/repopulation experiments, we provided the proof-of-concept evidence that resident CSCs are necessary and sufficient for the regeneration and repair of myocardial damage^21^.

In contrast to our data, Wallner et al.^22^ reported that ISO caused minimal myocardial necrosis, which recovered without any meaningful contribution of “c-Kit(+)-CSC-derived CM regeneration.” These results obtained using the c-kit^Cre^KI mutant mice^10,22^ have raised further scepticism about the role of CSCs in the adult heart.

Here we show that c-kit haploinsufficiency in c-kit^Cre^ KI mice impairs the endogenous cardioregenerative response after ISO affecting CSC activation and CM replacement. Repopulation of c-kit haploinsufficient myocardial tissue with wild-type CSCs, as well c-kit gene deficit correction of haploinsufficient CSCs, restores CM replacement and cardiac function after ISO. Thus, these data show that adult cardiomyogenesis depends on a diploid level of c-kit in CSCs.

## Materials and methods

### Animals

All animal experimental procedures were approved by Magna Graecia Institutional Review Boards on Animal Use and Welfare and performed according to the Guide for the Care and Use of Laboratory Animals from directive 2010/63/EU of the European Parliament. All animals received human care and all efforts were made to minimize animal suffering.

Mice were housed under controlled conditions of 25 °C, 50% relative humidity, and a 12 h light (6:00–18:00 h) and 12 h dark cycle, with water and food available ad libitum, except for tamoxifen (TAM) diet described below. Mice were anaesthetised by intraperitoneal (i.p.) injection of ketamine (100 mg/Kg) and xylazine (5 mg/Kg) or inhaled isoflurane (isoflurane 1.5% oxygen 98.5%, Iso-Vet, Healthcare). As wild-type animals, C57BL/6J mice were used (Jackson Labs, stock number 000664). c-kit^CreGFPnls/+^ and c-kit^MCM/+^ were provided by Dr. Jeffrey D. Molkentin (Cincinnati Children’s Hospital Medical Center, Cincinnati, OH)^10^. Heterozygous c-kit^CreGFPnls^ mice (abbreviated as c-kit^Cre/+^) were crossed with homozygous B6.Cg-Gt(ROSA)26Sor^tm9(CAG-tdTomato)Hze/J^ reporter mice (abbreviated as R26^floxed-stop-dTomato^ or R26^dT^, Jackson Labs, stock number 007909), to generate double heterozygous animals (abbreviated as c-kit^Cre/+^:R26^dT/+^) for experimental purposes. Heterozygous R26^dT/+^ mice were used as controls.

To assess CM replenishment by CSCs after ISO, Tg(Myh6-Cre/Esr1)1Jmk/J male mice (abbreviated as Tg-myh6^MCM^, Jackson Labs, stock number 005650) were crossed with homozygous Gt(ROSA)26Sor^tm4(ACTB-tdTomato,-EGFP)Luo^/J reporter mice (abbreviated as R26^mT/mG^or R26^mt-mg^, Jackson Labs, stock number 007576), to generate double heterozygous animals. These double mutant mice carry a TAM-inducible Cre Recombinase driven by the cardiac α-myosin heavy chain (α-MHC or myh6) promoter and a double fluorescent (dT/mGFP) gene reporter in the ROSA26 locus where, upon TAM-induced Cre-dependent recombination, a membrane-bound dimeric tomato (dT) is replaced by a membrane-bound green fluorescent protein (mGFP). Eight-week-old double heterozygous Tg-myh6^MCM^:R26^mT/mG^ male mice were fed for 4 weeks with 40 mg/Kg bw/d of TAM citrate chow (400 mg/Kg diet, ENVIGO, TD.130860) or alternatively were i.p. injected in alternate days with (Z)-4-Hydroxytamoxifen (Sigma, H7904) at the dose of 40 mg/Kg for 2 weeks and then used 2 weeks later. For each experimental procedure with TAM diet/injection, double heterozygous Tg-myh6^MCM^:R26^mT/mG^, Tg-myh6^MCM^ and R26^mT/mG^ heterozygous littermates mice, were fed with standard normal mouse diet as controls.

The final *n*-value for each experimental group is specified in the relative figure legends.

### ISO administration

ISO (Sigma-Aldrich #I6504; St. Louis, MO) was prepared by dissolving a desired amount of powder in NaCl 0.9%. The solution obtained was protected from the light and kept on ice until the injections. Before any ISO/Saline injection, the body weight of animals was determined, mice were anaesthetised using isoflurane and baseline echocardiography obtained. Then, mice were randomly divided in the different groups and on awakening prepared to receive saline or ISO at dose of 200 mg/Kg or 400 mg/Kg. The solutions were injected s.c. under the inter-scapular skin. All ISO injections were administered to male, 12-week-old mice of the specified strains.

### Echocardiography

Prior to echocardiography, mice were anaesthetised with isoflurane. Unconscious mice were weighed and secured in a supine position on a temperature-controlled restraining board. Anesthesia was maintained with 1–2% isoflurane in oxygen delivered through a nose cone. Four-limb lead electrocardiograms (Vevo 3100 and MP150, Biopac, Goleta, CA, USA) were simultaneously recorded. All hair in the thoracic region was removed using a depilatory agent and the area was cleaned with water. Ultrasound gel was applied to the thoracic region to improve sound wave transmission. All mice were maintained at heart rates > 400 b.p.m., while images were recorded. Echocardiographic images were obtained with a Vevo 3100 system (Visualsonics, Inc., Toronto, Canada) equipped with a MX550D ultra-high frequency linear array transducer (22–55 MHz). The transducer was positioned in a stationary stand perpendicular to the mouse (in some cases, manual adaptations were needed for optimal imaging). In brief, a frame rate of > 200 frames per minute was maintained for all B-mode and M-mode images. B-mode long-axis parasternal images were recorded when optimal views of the aorta, papillary muscle, and endocardium were visible. M-mode short-axis images were recorded at the level of the papillary muscles and the LV was bisected to obtain the optimal M-mode selection. Conventional echocardiographic measurements of the LV included ejection fraction (EF), fractional shortening (FS), end-diastolic dimension, end-systolic dimension, anterior and posterior wall thickness, and mass were obtained. For long-axis B-mode measurements, the endocardium was traced semi-automatically beginning from the mitral valve and excluding the papillary muscle. EF and FS were calculated by software using standard computational methods. Advanced cardiac analysis (regional and global cardiac measurements) were assessed by speckle-tracking echocardiography (Vevo LAB analysis software; VisualSonics). Cardiac cycles were acquired digitally from the parasternal long-axis and mid-ventricular short-axis views for the assessment of radial, circumferential, and longitudinal systolic strain/velocity (in accordance with myocardial fibre orientation at varying levels of the LV wall) and time-to-peak systolic strain/velocity. Images selected for strain analysis had well-defined endocardium and epicardium borders and no substantial image artefacts. Image analysis was performed according to the manufacturer’s instructions. The endocardium and epicardium were traced semi-automatically using VevoStrain software. The traces were manually adjusted to ensure adequate tracking of endocardium and epicardium borders. Velocity, displacement, strain, and strain rate were calculated for radial and longitudinal planes. In long axis, the basal anterior-septum, mid-anterior-septum, apical anterior-septum, basal posterior wall, mid-posterior wall, and apical posterior segments were defined. In mid-ventricular short axis, the anterior, anterior-septum, inferior-septum, inferior, posterior, and anterior-lateral segments were further delineated. Tissue contraction patterns were expressed as negative strain values for longitudinal and circumferential motion, and positive values for radial strain. In each segment, peak systolic strain (%) and time-to-peak systolic strain (ms) were analysed. Global average peak values for circumferential and longitudinal strain are reported.

The *n*-value for each experimental group is specified in the relative figure legends.

### Myocyte necrosis analysis

To assess CM necrosis, 12-week-old male mice were used. Mice (strains are specified in the main text and figure legends) received i.p. injection of 100 µg/100 µl of a monoclonal antibody against cardiac myosin (MF-20, ID: AB_2147781, DSHB) 2 h after ISO injection. Alternatively, mice received i.p. injections of 50 µl/10 g of the body weight of a 20 mg/ml stock solution of Evan’s blue dye (EBD, Sigma-Aldrich) dissolved in NaCl 0.9% administrated 24 h before ISO/Saline injections. All animals were sacrified at 1 day after ISO/Saline injection and the heart fixed with 4% paraformaldehyde (PFA).

The *n*-value for each experimental group is specified in the relative figure legends.

### Cardiac troponin analysis

To assess the presence of myocyte necrosis induced by a single dose of ISO, the levels of high-sensitive cardiac Troponin T (cTnT) in the blood serum were analysed 1 day after ISO/Saline. Animals were anaesthetized (ketamine 100 mg/Kg and xylazine 5 mg/Kg) and 1 ml of blood was taken from the orbital sinus inserting the tip of a fine-glass Pasteur pipette into the corner of the eye underneath the eyeball, directing the tip at a 45° angle towards the middle of the eye. The blood was collected in a vacutainer (Vacuette Tube 3 ml K3E K3EDTA, # 454086). The threshold for cTnT positivity in mice was established at the 99th percentile of cTnT values in blood samples from 15 consecutive control mice, using a commercially available and clinically validated blood cTnT kit. The *n*-value for each experimental group is specified in the relative figure legends.

### BrdU and Edu incorporation in vivo

To detect in vivo cell proliferation after ISO administration, 12-week-old male C57BL/6J mice were injected at 12 pm with saline or ISO at dose of 200 mg/Kg. Six hours later, mice received an i.p. injection of Bromodeoxyuridine (BrdU) or 5-Ethynyl-2´-deoxyuridine (EdU) at a concentration of 50 mg/Kg in 100 µl of a 50% de-ionized water and 50% dimethylsulfoxide (DMSO) solution (Brdu, Sigma B9285; EdU, Life Technologies E10187). The morning after, all mice were anaesthetized using isoflurane and implanted subcutaneously (between the two scapulae) with mini-osmotic pumps (ALZET)^21^ to systemically release BrdU or EdU (50 mg/Kg/Day both) for 7 or 28 days prepared by dissolving the powders in 50% de-ionized water and 50% DMSO. Mice from each group were sacrified at 28 days after ISO/Saline administration and the hearts were fixed in formalin for immunohistochemistry analysis.

To assess the regenerative potential of c-kit^Cre^ mice, heterozygous c-kit^Cre/+^ mice, heterozygous R26^dT/+^ mice, and double heterozygous c-kit^Cre/+^:R26^dT/+^ mice were treated with ISO/Saline and implanted for 28 days with BrdU pumps as described above. In addition, ~35% of available male c-kit^Cre/+^:R26^dT/+^ exhibited spontaneous alterations of cardiac function at baseline pre-ISO injections and had to be excluded. To assess the effects of ISO administration on activation of quiescent resident CSCs, R26^dT/+^ and c-kit^Cre/+^:R26^dT/+^ mice were divided in saline-injected and ISO-injected groups as above. BrdU, at a concentration of 50 mg/Kg/Day, was i.p. administered in vivo for 3 days in adult mice every 12 h before sacrifice. The mice were sacrified at 1 day or 3 days after ISO injection and hearts were dissociated to obtain a myocyte-depleted cardiac cell preparation for fluorescence-activated cell sorting (FACS) analysis as described^18,21,23^. To further assess the cardiac generation process, myocardial nuclei were isolated 28 days after ISO or Saline from mouse hearts continuously labelled with BrdU and evaluated by FACS analysis for BrdU detection as previously reported^24^.

The *n*-value for each experimental group is specified in the relative figure legends.

### ISO + 5-FU-induced cardiomyopathy

Chronic HF was induced in 12-week-old male C57BL/6J mice, in heterozygous Tg-myh6^MCM^ mice, and in Tg-myh6^MCM^: R26^mT-mG/+^ double heterozygous mice by a subcutaneous single-dose administration of ISO 200 mg/Kg followed at 72 h by systemic administration of 5-Fluorouracil (5-FU,15 mg/Kg/daily) for 25 days through subcutaneously implantation of mini-osmotic pumps. Before pumps implantation the mice were anaesthetized using isoflurane. Twenty-eight days after ISO injection, 5-FU-realizing mini-pumps were removed. Tg-myh6^MCM^: R26^mT-mG/+^ double heterozygous mice were killed and the hearts were fixed in 4% PFA for immunohistochemistry analysis. C57BL/6J mice were randomly divided to receive tail vein CSCs/Saline injections as indicated. The animals were sacrified 28 days after CSCs/Saline injections and the hearts were fixed in formalin for immunohistochemistry analysis or dissociated to obtain a cardiac cell preparation for FACS analysis.

The *n*-value for each experimental group is specified in the relative figure legends.

### Myocyte-depleted cardiac cell isolation

CSCs were isolated from the relative adult mouse hearts by enzymatic dissociation using a Langerdoff-modified apparatus^23^ or using gentle MACS Dissociator (Miltenyi Biotec)^17,18^. Briefly, for the Langerdoff-modified apparatus, the heart was excised, the aorta cannulated, and hung on a retrograde perfusion system. Heart was perfused with collagenase type II dissolved HEPES-MEM (Sigma) (Worthington) at 37 °C with 85% O_2_ and 15% N_2_, then the heart was removed from the apparatus, cut into small pieces, and the fragments shaken in re-suspension medium at 37 °C. CMs and myocyte-depleted small cardiac cells were separated by centrifugation. For gentle MACS- isolation, manufacturer instructions were followed to obtain myocyte-depleted cardiac small cells for FACS analysis. To obtain CSC-enriched CD45^neg^CD31^neg^c-kit^pos^ cells, the MACS technology was used with direct CD45- and CD31-negative and then c-kit-positive specific anti-mouse microbeads sorting (Miltenyi Biotec)^17,18,23^.

### Cell cultures and transfection

Isolated CSC-enriched CD45^neg^CD31^neg^c-kit^pos^ cardiac cells were plated in gelatin-coated dishes in CSC growth medium containing Dulbecco’s modified Eagle’s medium-F12Ham’s (Gibco, Life Technologies) with insulin-transferrin-selenium (1%, Life Technologies), epidermal growth factor (final medium concentration: 20 ng/ml, Peprotech), basal fibroblast growth factor (final medium concentration: 10 ng/ml, Peprotech), and leukemia inhibitory factor (final medium concentration: 10 ng/ml, Miltenyi), and 1:1 ratio of Neurobasal medium (Gibco, Life Technologies) containing 37 mg of l-glutamine, B27 supplement (2%, Life Technologies) and N2 supplement (1%, Life Technologies), penicillin–streptomycin (1%, Life Technologies), Fungizone (0.1%, Life Technologies), and gentamicin (0.1%, Life Technologies) sterilized through a 0.22 µm pore filter into a sterile container, and store it at 4 °C for up to 3 weeks^21,23^. The CSC growth medium was supplemented with 10% ESQ-FBS (Life Technologies). Cells were maintained at 37 °C in ambient O_2_ (21%) and 5% CO_2_. Media were replenished every 48 h and cells were passaged at a 1:4 ratio. dT-wtCSCs, dT-W^Cre^CSCs, and YFP-wtCSCs (also CSC^YFP^) clones were respectively obtained from R26^mT-mG/+^, c-kit^CreER(T2)/+^:R26^mT-mG/+^^18^, and R26^stop^^YFP^^21^ mice by depositing through serial dilution a single CD45^neg^CD31^neg^c-kit^pos^ isolated cardiac cells from mice heart into 96-well gelatin-coated Terasaki plates. Individual clones were identified, expanded, and grown in CSC growth medium. Cell growth of freshly isolated (P1) W^Cre^CSCs vs. wtCSCs was assessed by plating 1 × 10^4^ cells in gelatin-coated dishes in CSC growth medium. Cells were counted every 24 h until day 4. Cardiac fibroblasts (cFBs) were isolated from R26^stopYFP^ mice as previously described^21^. The Cre-reporter gene, Yellow fluorescent protein (YFP), was switched on through transduction of R26^stopYFP^ CSCs and cFBs with an Adeno-CMV-Cre in vitro (Santa Cruz Biotechnology) as per the manufacturer’s instructions. Stealth RNAi c-KIT small interfering RNA (Thermo Fisher Scientific) was transfected in CSC^YFP^ using Lipofectamine RNAiMAX Transfection Reagent (Thermo Fisher Scientific) and efficient KIT knockdown was verified by reverse transcriptase-PCR as per the manufacturer’s instructions.

The *n*-value of biological replicates is specified in the relative figure legends.

### BAC c-kit vector generation and transfection in W^Cre^CSCs

A purified bacterial artificial chromosome (BAC), BAC^c-kit^ DNA was generated and then transfected in a dT-W^Cre^CSCs clone obtained from c-kit^CreER(T2)/+^:R26^mT-mG/+^ mice as previously described^18,25^. A single-cell-derived sub-clone of dT-W^Cre^CSCs containing a single BAC^c-kit^ copy was obtained as previously described and used for the in vivo cell transplantation experiments^18^.

### MethoCult assay in vitro

Colony Forming Unit (CFU) formation from cloned wtCSCs vs. W^Cre^CSCs was assessed by plating relative cells in 1.2 ml of MethoCult medium (STEMCELL Technologies, Vancouver, Canada) (1% methylcellulose in CSC growth medium). Colonies were counted at 14 days after plating. The *n*-value for the biological replicates is specified in the relative figure legends.

### Bone marrow cell isolation

Bone marrow (BM) cells were isolated from mice by flushing both femurs and tibias with phosphate-buffered saline (w/o Ca, w/o Mg) using a 26-gauge needle syringe. Cells were pelleted, washed, and re-suspended for FACS analysis. For BM lineage-negative FACS analysis, a Lineage Negative Depletion Kit was used (Miltenyi Biotec). The *n*-value of biological replicates is specified in the relative figure legends.

### Tissue harvesting, histology, and immunohistochemistry

For immunohistochemistry analysis, the abdominal aorta was cannulated and the heart arrested in diastole using cadmium chloride/potassium solution. The animals were perfused through the cannulated aorta and fixed with 10% buffered formalin or with 4% PFA. The hearts were cut into apical, mid, and basal regions, and the right and left atria. After being weighed, the LV was sectioned embedded in paraffin or in Optimal Cutting Temperature Compound. Tissues were cut in 5 µm or 10 µm cross-sections, respectively. Apoptotic cells on cross-sections were detected using the primary antibody caspase-3 (1:100 dilution: Abcam) and horseradish peroxidase-conjugated secondary antibody, and visualized by 3,3′-Diaminobenzidine (DAB) substrate histochemistry. Sections were stained with hematoxylin and eosin (H&E) following standard procedures. Formalin-fixed sections were stained with Masson’s Trichome (PolySciences, Inc.) for bioquantification of fibrosis^26^. CM cross-sectional area was measured through immunostaining with Wheat Germ Agglutinin (WGA) Alexa Fluor 647 conjugate (1:200 dilution; Invitrogen) and digital analysis of acquired cardiac cross-section images (Leica, 1128 LAS AF Software). CM diameter was measured across the nucleus on three transverse sections (~500 myocytes/animal were sampled). For immunostaining and BrdU detection, antigen retrieval was achieved using Target Retrieval Solution, Citrate pH 6 (DAKO). For EdU detection, the Click-iT EdU Imaging Kit (Life Technologies) was used. The following primary antibodies were used: anti-BrdU (1:50 dilution; Sigma), anti-EdU (1:500, Click-iT EdU Imaging Kit, Life Technologies), monoclonal antibody against cardiac myosin (MF-20, ID: AB_2147781, DSHB), anti-Actinin (1:50 dilution; Santa Cruz), anti-Cardiac Troponin I (1:50 dilution; Abcam), anti-GFP (for YFP detection) (1:50 dilution; Rockland), anti-RFP (for dTomato detection) (1:50 dilution; Rockland), anti-c-kit (1:50 dilution; R&D Systems), and anti-pericentriolar material 1 (PCM-1) (1:200 dilution; Atlas Antibodies). The primary antibody was revealed by respective anti-mouse IgG, anti-rabbit IgG, or anti-goat IgG secondary antibody (1:100 dilution; Jackson Immunoresearch). The nuclei were counterstained with the DNA binding dye, DAPI (4, 6-diamidino-2-phenylindole, Sigma) at 1 µg/ml. BrdU and EdU fluorescence quantification has been obtained through manual counting of respective histologic samples and the number of BrdU^pos^ or EdU^pos^ CMs was expressed as a percent fraction of the total CM nuclei^17,21^. To evaluate the CM progeny of the YFP^pos^ or dTomato^pos^ CSCs in vivo, the number of double positive YFP^pos^/actinin^pos^ (or YFP^pos^/cTnI^pos^) and dTomato^pos^/actinin^pos^ (or dTomato^pos^/cTnI^pos^) CMs were counted in cardiac cross-sections derived from mice for each power field using a ×63 objective for a total of 20 fields^17,21^. The number of c-kit^pos^CD45^neg^CD31^neg^ cells in all experimental group was expressed per mm^2^. The number of YFP^pos^ or dTomato^pos^ CMs in all experimental group was expressed as a percent fraction of the total CM number per mm^2^. The number of necrotic/dead MF-20^pos^ and EBD^pos^ (the latter fluoresces in red) CMs was manually counted in cardiac cross-sections for each power field using a ×63 objective for a total of 20 fields^17,21^, and the number of MF-20^pos^ and EBD^pos^ CMs was expressed as a percent fraction of the total CM number per mm^2^. All stainings were acquired and analysed using confocal microscopy (LEICA TCS SP5 and SP8).

### Flow cytometry

Cell analysis on total freshly isolated c-kit^pos^ cardiac cells, freshly isolated CD45^neg^CD31^neg^c-kit^pos^ CSCs, and BM cells was performed using a FACSCanto II (BD Biosciences) or a BD FACSAria™ III (BD Biosciences). FlowJo software (Treestar) was used to identify the percentage of cells expressing the different cell surface markers of interest. Voltages for cytometry acquisition were determined using single stain. Specific antibodies used are shown in Supplementary Table 1 (bottom of this file). Appropriate labelled isotype controls were used to define the specific gates. The *n*-value for each group is specified in the relative figure legends.

### Statistical analysis

Statistical analysis was performed with GraphPad Prism version 6.00 for Macintosh (GraphPad Software). Quantitative data are reported as mean ± SD and binary data by counts. Significance between two groups was determined by Student’s *t*-test or paired *t*-test as appropriate. For comparison between multiple groups, analysis of variance was used. A *P*-value < 0.05 was considered significant. Tukey’s post-hoc method was used to locate the differences. In these cases, the Type 1 error (*α* = 0.05) was corrected by the number of statistical comparisons performed. For the in vitro cell and molecular biology experiments, the Kruskal–Wallis test (for multiple‐group comparison) and the Mann–Whitney *U*-test (for comparison between two groups) were also performed.

## Results

### Acute ISO overdose causes CM necrosis and apoptosis in a dose-dependent manner in the LV sub-endocardial apex

**Fig. 1:**
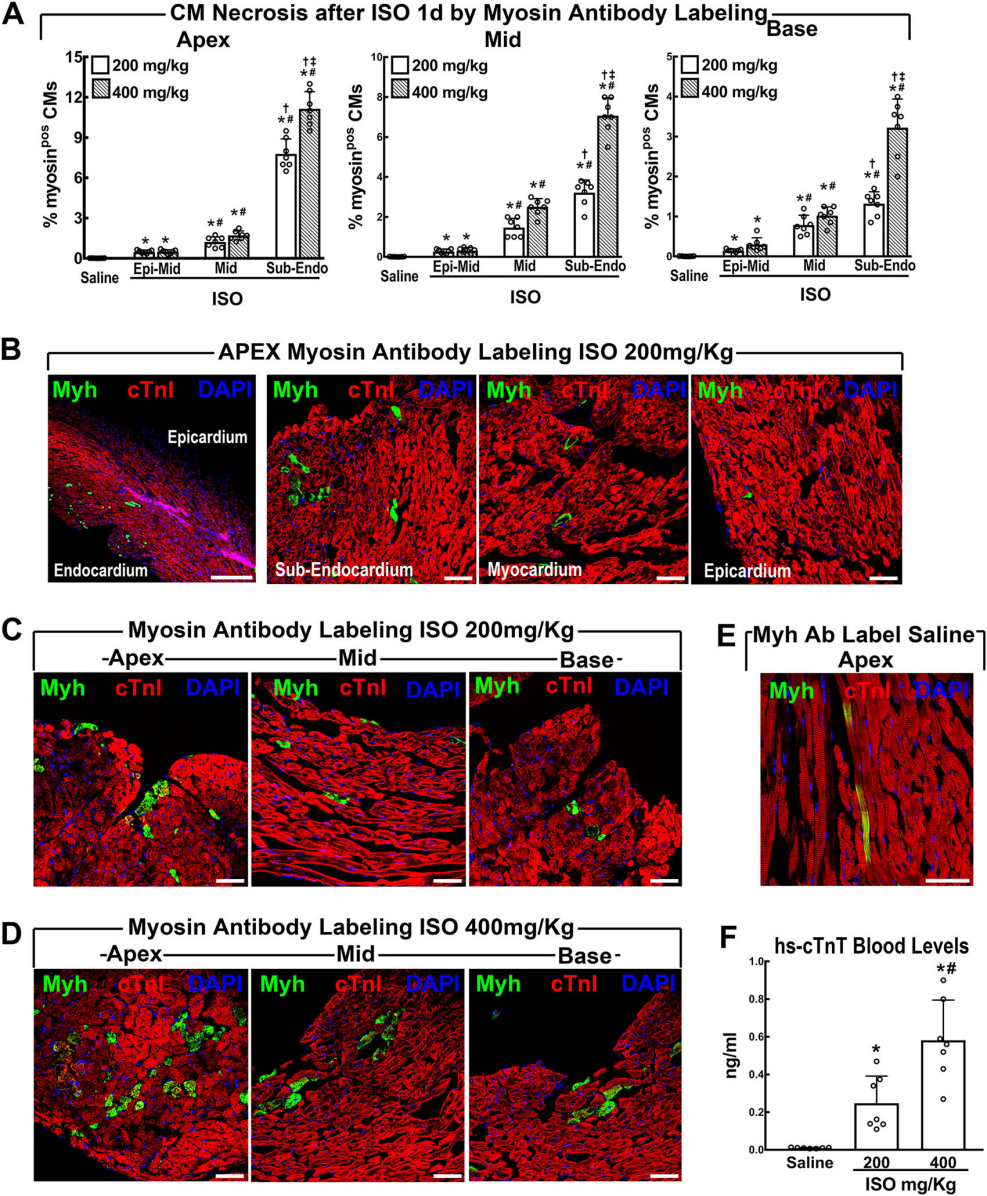
ISO acutely causes cardiomyocyte necrosis mainly targeting the sub-endocardial apex. **a** The fraction of necrotic cardiomyocytes (CM) was significantly increased in a dose-dependent manner at 1 day after ISO (200 and 400 mg/Kg) with higher frequency in the Apex sub-endocardium. *n* = 7 per group; *p < 0.05 vs. saline; #*p* < 0.05 vs. Epi; †*p* < 0.05 vs. Mid; ‡*p* < 0.05 vs. 200 mg/Kg (one-way ANOVA analysis with Tukey’s multiple comparison test). **b** Representative images of necrotic CMs labelled in vivo with anti-myosin antibody (green) showing higher damage in the sub-endocardium when compared with myocardium and epicardium. **c**, **d** Representative images of necrotic CMs labelled in vivo with anti-myosin antibody (green) showing ISO dose-dependent higher damage in the apex when compared with mid and base in LV regions 1 day after ISO 200 (**c**) or 400 (**d**) mg/Kg, respectively. **e** Representative image of a non-necrotic anti-myosin antibody (green)-labelled normal cardiomyocyte. **f** Plasma cTnI was significantly elevated in a dose-dependent manner at 1 day after ISO. hs-TnT = high-sensitive cardiac Troponin T. *n* = 7 per group; *P < 0.05 vs. Saline; #*p* < 0.05 vs. 200 mg/Kg (one-way ANOVA analysis with Tukey’s multiple comparison test). Scale bars = 50 µm except for **b** left panel = 300 µm. All data are mean ± SD

ISO at 200 mg/Kg^21^ (*n* = 14) or at 400 mg/Kg (lethal dose in the study by Wallner et al.^22^) (*n* = 14) caused diffuse CM necrosis in 12-week-old C57BL/6J male mice as revealed by in vivo myosin antibody labelling^27^ 1 day after ISO injection (Fig. 1). Myosin-labelled CMs showed clear morphologic features of necrosis with loss of cell membrane integrity and architectural disarray (Fig. 1). The damage was distributed in an intensity/density progressive gradient from epicardium to sub-endocardium and from base to apex (Fig. 1a–c). CM death mostly concentrated to the sub-endocardial apex where myosin-labelled necrotic CMs reached up to ~8% and ~12% after ISO 200 or 400 mg/Kg ISO, respectively (Fig. 1a–d). Only very rare (~0,001% of total) myosin-labelled CMs were detected in the saline-injected mice; however, these anti-myosin-labelled CMs were always normally shaped and without additional signs of necrosis (Fig. 1e). Increased cTnT blood levels independently confirmed CM necrosis directly proportional to the ISO dose (Fig. 1f). The normal cTnT values baseline was established at <0.01 ng/ml in 15 consecutive control mice. Blood cTnT levels increased to 0.25 ± 0.14 and 0.58 ± 0.21 ng/ml 1 day after 200 or 400 mg/Kg ISO, respectively (Fig. 1f).

The assessment of EBD^28^-positive CMs at 1 day after ISO closely reproduced the CM necrosis data obtained with myosin antibody labelling (Supplementary Fig. 1). In addition, CM necrotic death after ISO was also evident by H&E histochemistry (Supplementary Fig. 1B). Finally, ISO exposure caused apoptotic CM death in a dose-dependent manner as identified by caspase-3 labelling (Supplementary Fig. 1C).

### High dose of ISO transiently impairs LV regional performance

To assess the functional consequences of ISO-induced CM damage and death, additional mice were injected with either 200 mg/Kg or 400 mg/Kg ISO and analysed by echocardiography (ECHO). Saline-injected mice were used as sham controls. Two mice in the 200 mg/Kg and 3 mice in the 400 mg/Kg ISO group died during the first week.

**Fig. 2:**
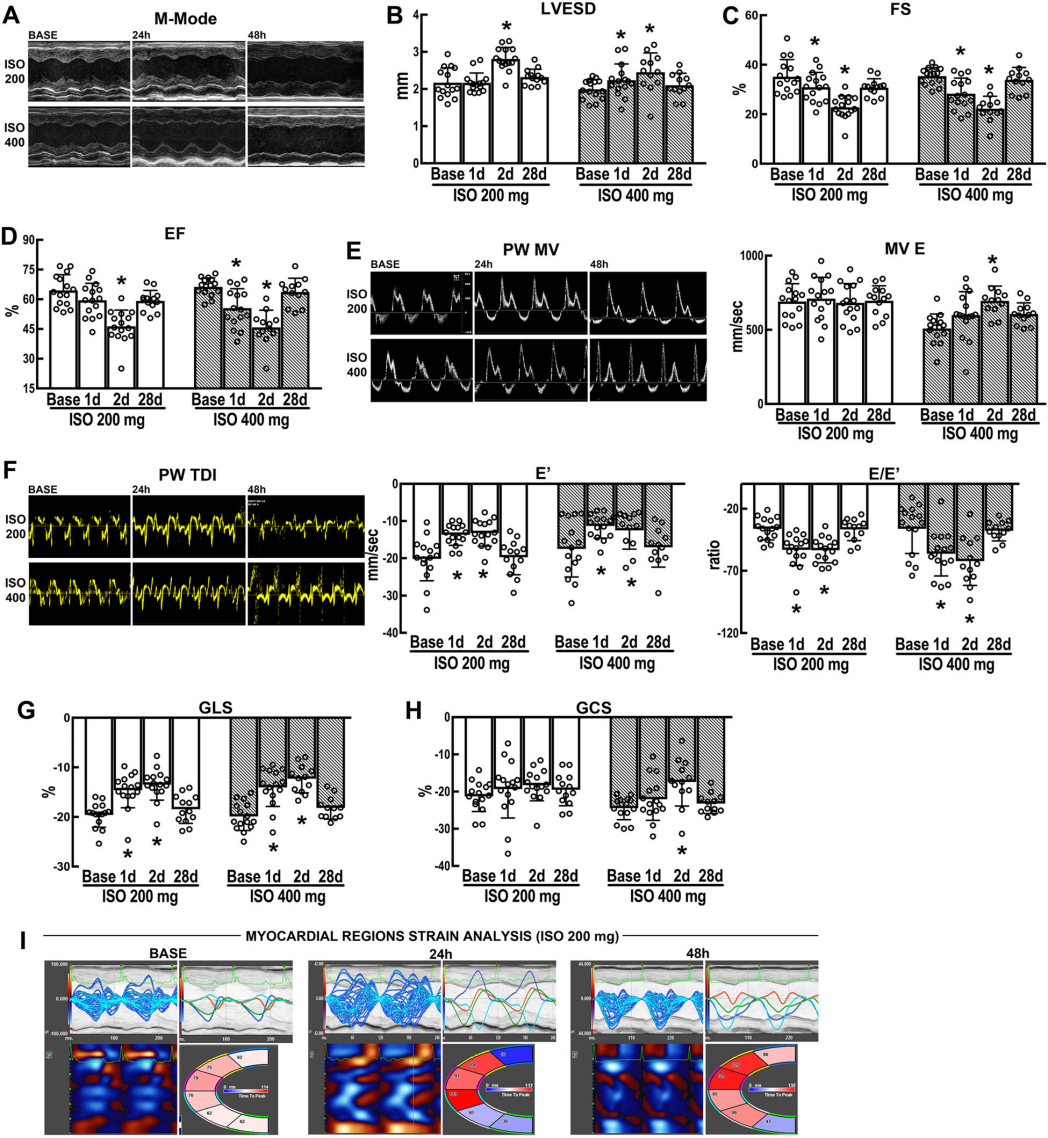
Isoproterenol transiently impairs cardiac function. **a** Representative M-mode tracing at Base, 1 day and 2 days after a single s.c. injection of Isoproterenol (ISO) at 200 or 400 mg/Kg. **b**–**d** Cumulative data of cardiac dimensions and function at the different time points. LVESD = left ventricular end-systolic diameter (**b**), FS = fractional shortening (**c**), and EF = ejection fraction (**d**). **b**, **d** *p < 0.05 vs. Base, 1d and 28d in ISO 200 mg; *p < 0.05 vs. Base and 28d in ISO 400 mg (one-way ANOVA analysis with Tukey’s multiple comparison test). **c** *p < 0.05 vs. Base and 28d in ISO 200 mg and *p < 0.05 vs. Base and 28d in ISO 400 mg (one-way ANOVA analysis with Tukey’s multiple comparison test). **e** Representative pulsed wave Doppler Mitral Velocity tracing (PW MV, left) and cumulative transmitral Doppler early filling velocity (MV E data, right) at Base, 1d and 2d after a single s.c. injection of ISO at 200 or 400 mg/Kg. *p < 0.05 vs. Base, 1d, and 28d in ISO 400 mg (one-way ANOVA analysis with Tukey’s multiple comparison test). **f** Representative pulsed wave tissue Doppler imaging velocity tracing (PW TDI, left) and cumulative early diastolic mitral annular velocity value (*E*’, mid) and *E*/*E*’ ratio (right) data at Base, 1d and 2d after a single s.c. injection of ISO at 200 or 400 mg/Kg. *p < 0.05 vs. Base and 28d in ISO 200 mg and *p < 0.05 vs. Base and 28d in ISO 400 mg (one-way ANOVA analysis with Tukey’s multiple comparison test). **g**, **h** Cumulative data of global longitudinal and circumferential strain (GLS and GCS, respectively) at the different time points at Base, 1d and 2d after a single s.c. injection of ISO at 200 or 400 mg/Kg. **g** *p < 0.05 vs. Base and 28d in ISO 200 mg and *p < 0.05 vs. Base and 28d in ISO 400 mg (one-way ANOVA analysis with Tukey’s multiple comparison test). **h** *p < 0.05 vs. Base, 1d, and 28d in ISO 400 mg (one-way ANOVA analysis with Tukey’s multiple comparison test). **i** Representative images of regional speckle-tracking strain analysis throughout four segments (apical, anterior and inferior segments wall, and mid-anterior and inferior wall) at Base, 1d and 2d after a single s.c. injection of ISO at 200 mg/Kg.  n= 15 (ISO 200 mg/Kg, BASE, 1d, and 2d); *n* = 13 (ISO 200 mg/Kg, 28d); *n* = 15 (ISO 400 mg/Kg, BASE and 1d); *n* = 12 (ISO 400 mg/Kg, 2d, and 28d). All data are mean ± SD

Only ISO at 400 mg/Kg significantly decreased LV EF and FS after 1 day compared with baseline. There was a nonsignificant decrease in EF in mice treated with 200 mg/Kg ISO (Fig. 2a–d, Supplementary Fig. 2, and Supplementary Table 2). However, despite a preserved EF (Fig. 2d and Supplementary Table 2), ISO 200 mg/Kg mice showed a significant decrease in FS at 1 day (Fig. 2c and Supplementary Table 2). EF 2 days after 200 mg/Kg ISO showed a significant depression compared with baseline. The latter could be explained by the evidence that a detectable reduction in EF is often a late phenomenon^29^ (Fig. 2d and Supplementary Table 2). At the same time point, LV function was also consistently depressed in mice treated with 400 mg/Kg ISO (Fig. 2a–d and Supplementary Table 2). Segmental dysfunction was more prominent at mid-apical regions (see below) and evident apical ballooning was present in approximately a third of the injected mice.

Diastolic dysfunction was evident at 1 and 2 days after ISO, both at 200 or 400 mg/Kg, when compared with base (Fig. 2e, f, Supplementary Fig. 2, and Supplementary Table 2). Specifically, the diastolic function shows that the *E*’-value decreases (indicating a reduction of LV longitudinal myocardial relaxation) (Fig. 2f and Supplementary Table 2), whereas *E*/*E*’ (the ratio of transmitral Doppler early-filling velocity to tissue Doppler early-diastolic mitral annular velocity, an index of LV end-diastolic filling pressure) significantly increases (Fig. 2f and Supplementary Table 2). All these parameters had returned to baseline at 28 days after ISO (Fig. 2a–f and Supplementary Table 2).

Global strain analysis^29^ was able to detect subtle changes in cardiac performance earlier than conventional standard echocardiographic measures in 200 mg/ Kg ISO-treated mice (Fig. 2g, h and Supplementary Table 2). ISO, both at 200 and 400 mg/Kg, significantly decreased the value of global longitudinal strain at 1 day compared with baseline. Global longitudinal strain remained consistently depressed at 2 days (Fig. 2g and Supplementary Table 2). Interestingly, only in mice treated with 400 mg/Kg ISO, global circumferential strain was significantly decreased at 2 days compared with baseline (Fig. 2h and Supplementary Table 2). All strain values were normalized at 28 days (Fig. 2g, h and Supplementary Table 2).

Finally, regional speckle-tracking strain analysis throughout four segments at 1 day after ISO 200 mg/Kg showed an increase in time-to peak value in apical segments (even in mice with preserved EF) when compared with the uninjured segments (Fig. 2i and Supplementary Table 2). Also, segments analysis of wall motion abnormalities and wall synchronicity detected early cardiac damage (Fig. 2i and Supplementary Table 2).

### CM loss by a single high dose of ISO triggers their replacement with new CMs by the CSCs and not through division of pre-existing CMs

**Fig. 3:**
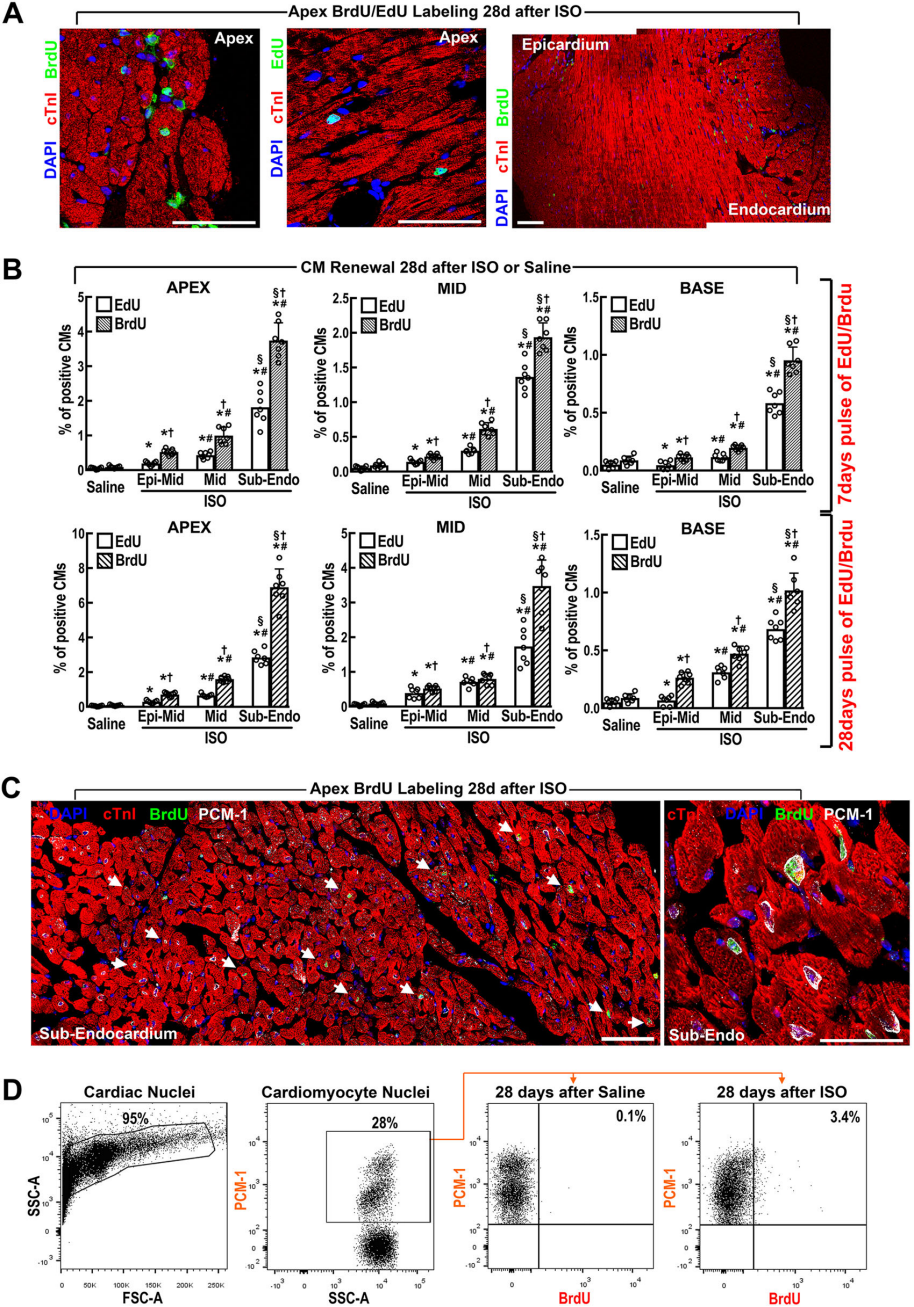
Cardiomyocyte Replenishment after ISO-induced CM loss. **a** Representative confocal microscopy images of BrdU (left) and EdU (middle) incorporation in the apical endocardium 28 days after ISO in wt C57BL/6J mice implanted with subcutaneous mini-pumps to systemically release BrdU or EdU for 28 days. Right, representative confocal microscopy of BrdU incorporation from epicardium to endocardium of the LV Apex 28 days after ISO in wt mice implanted with subcutaneous mini-pumps to systemically release BrdU for 28 days. It is evident the intensity gradient of BrdU incorporation from the epicardium to the endocardium. Scale bar = 50 μm. **b** Number of newly generated BrdU^pos^ or EdU^pos^ CMs at 7 and 28 days in saline or ISO-treated wt mice implanted with subcutaneous mini-pumps to systemically release EDU or BrdU for 7 or 28 days, respectively. *n* = 7 per group, *p < 0.05 vs. Saline, #*p* < 0.05 vs. Epi, §*p* < 0.05 vs. Myo, and †*p* < 0.05 vs. EdU (one-way ANOVA analysis with Tukey’s multiple comparison test). **c** Representative reconstruction of three overlapping confocal microscopy images (left) and a high magnification field (×3 zoom from a ×63 microscopy objective, right) showing newly formed cardiomyocytes (arrowheads) whose nuclei are unambiguously identified by PCM-1 (white fluorescence) while being BrdU-positive (green) 28 days after ISO injury in the apical sub-endocardium. DAPI (Blue) depicts cell nuclei. **d** Cardiac nuclei are identified by FACS analysis based on DAPI labelling (left panel). Fluorescent gating allows the separation of cardiomyocyte nuclei (PCM-1-positive) and non-cardiomyocyte (PCM-1-negative) nuclei in cardiac nuclei preparations isolated from digested heart tissue (left-mid panel). Representative plots show the number of BrdU-labelled cardiomyocyte nuclei (gated on PCM-1-positive nuclei) 28 after saline (right-mid panel) or Isoproterenol (ISO, right panel) injection. It should be noted that the number of BrdU^pos^ cardiomyocyte nuclei should be corrected when extrapolating it to number of cardiomyocytes considering that by the vast majority of adult cardiomyocytes in the adult murine hearts are binucleated. Scale bars = 50 µm. All data are mean ± SD

We assessed new CM formation 28 days after ISO by comparing head-to-head BrdU vs. EdU continuous labelling (through mini-pump implants releasing BrdU or EdU 50 mg/Kg daily) for 7 or 28 days after ISO (200 mg/Kg). We detected new CM formation both in EdU- and BrdU-continuously labelled mice, which was more intense and mainly localized to the sub-endocardial apex (Fig. 3a). The fraction of BrdU^pos^ and EdU^pos^ CMs was ~7% and ~3% of the CMs in the sub-endocardial apical layer at 28 days, respectively, compared with ~0,1% in saline-treated mice. Newly formed CMs were significantly less abundant in the mid- and basal myocardial regions (Fig. 3b), in agreement with less CM damage in these areas (Fig. 1). Also, BrdU^pos^ and EdU^pos^ CMs were significantly less abundant with only 7 days compared with 28 days continuous thymidine analogues’ administration after ISO (Fig. 3b). In all cases, however, EdU-labelled less (< 50%) newly formed CMs than BrdU either after 7-days or 28-days of continuous administration (Fig. 3b).

CM nuclei-specific identification with PCM-1 antibody^24^ show that the number of BrdU^pos^PCM-1^pos^cTnI^pos^ CMs (5.6 ± 1%, Fig. 3c) 28 days after ISO in the sub-endocardial layer of the apical section was practically undistinguishable from the number of BrdU^pos^ CM nuclei identified and counted by morphological features (Fig. 3b). Isolated CM nuclei were evaluated for PCM-1 and BrdU detection by FACS (Fig. 3d), allowing for unambiguous direct evaluation of BrdU^pos^ CMs. These data well reproduced the immunohistochemistry data in Fig. 3b.

We also analysed new CM formation using double transgenic myh6-mER-Cre-mER//R26R^mT-mG^ mice (abbreviated as Tg-myh6^MCM:^R26^mT-mG^ mice). Dilution of the GFP^pos^CMs/dTomato^pos^CMs ratio with an increase of dTomato^pos^ CMs after ISO (Supplementary Fig. 3) in these mice show that the new CM formation was not due to division of pre-existing CMs. Also, these data implicate that increased number of dTomato^pos^ CMs had to have arisen from non-myocyte myogenic progenitor/precursor cells^30–32^.

### c-kit receptor hemizygosity impairs the adult cardiac regenerative response after ISO

**Fig. 4:**
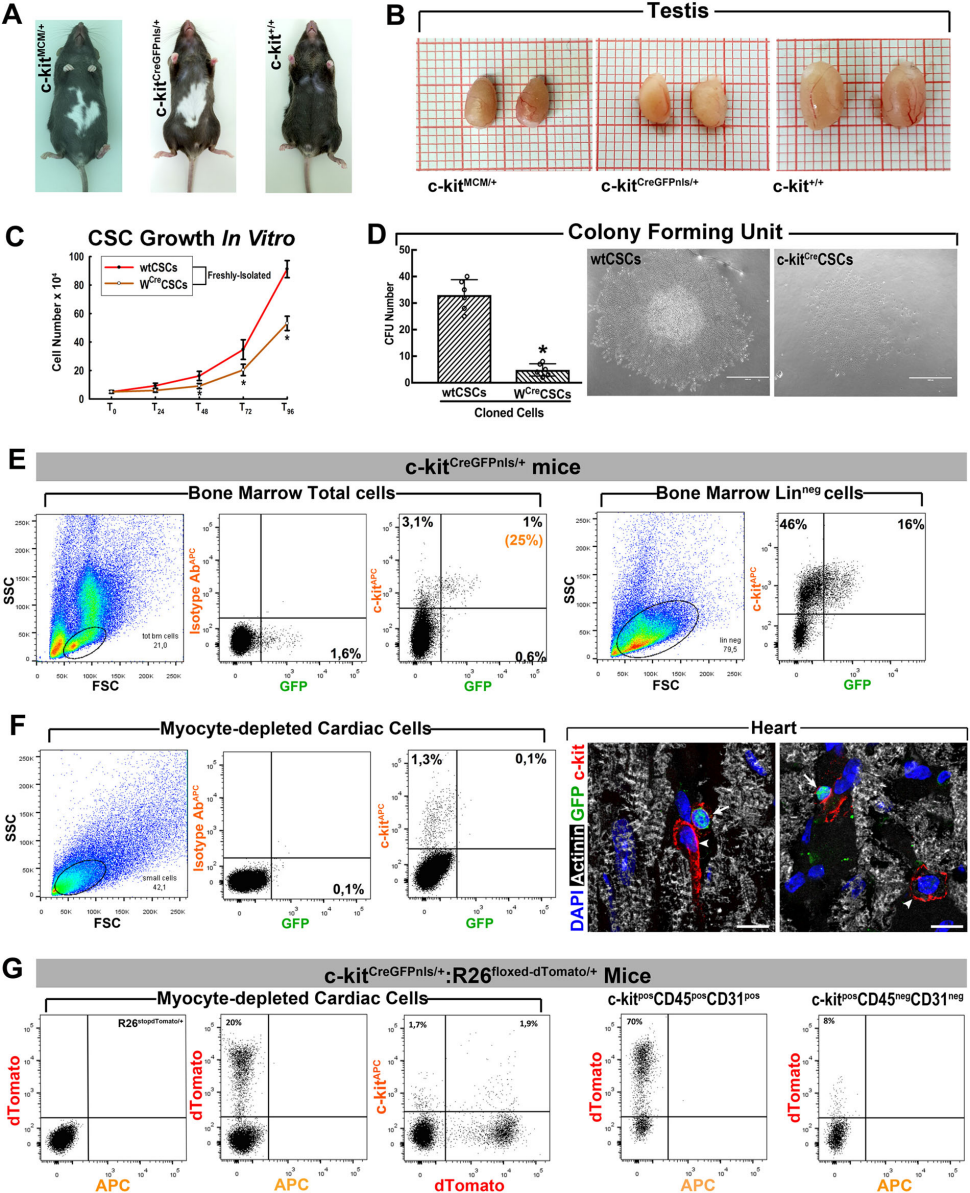
c-kit^Cre^ mice fail to label resident CSCs. **a** Photographs of representative adult c-kit^MCM^ and c-kit^CreGFPnls/+^ mice showing the typical W-mutation-determined piebald and white belly spot as compared with c-kit^+/+^ C57BL/6J mouse. **b** The testis from 2-month-old kit^MCM^ and c-kit^CreGFPnls/+^ show a significant size reduction compared to c-kit^+/+^ mice. **c** Cell growth curve of freshly isolated (P1) W^Cre^CSCs vs. wtCSCs over 96 h. *n* = 7 biological replicates, *p < 0.05 vs. wtCSCs (Kruskal–Wallis test). **d** Colony number of 100 cloned wtCSCs vs. W^Cre^CSCs in Methocult. *n* = 7 biological replicates, *p < 0.05 vs. wtCSCs (Student’s *t*-test). On the right, representative light microscopy images of a colony from wtCSCs and W^Cre^CSCs, respectively. *n* = 6 plots biological replicates. Scale bars = 1000 µm. **e** GFP expression (from the c-kit^CreGFPnls^ allele) in the monocyte–lymphocyte gate of total bone marrow (BM) cells population and in total c-kit^pos^ BM cells from 2-month-old c-kit^CreGFPnls/+^ mice. Less than 25% of the total c-kit^pos^ BM cells express GFP in c-kit^CreGFPnls/+^ mice. Concurrently, <35% of the lineage-negative (Lin^neg^) c-kit^pos^ BM cells (including HSCs) express GFP in c-kit^CreGFPnls/+^ mice. **f** GFP expression (from the c-kit^CreGFPnls^ allele) in cardiomyocyte-depleted cardiac cells (left) and in cardiac c-kit^pos^ cardiac cells (right) from 2-month-old c-kit^CreGFPnls/+^ mice. Less than 10% of these cells express GFP. Right, confocal microscopy representative images of cardiac cross-sections showing that GFP nuclear expression is expressed only in some (20 ± 3%) of the c-kit-expressing cardiac cells. Scale bar = 20 µm. (**e**, **f** representative of *n* = 5 BM/Hearts). **g** Mice KI-ed within the first exon of the c-kit locus to express Cre recombinase and GFP with a nuclear localization sequence (eGFPnls) behind an internal ribosome entry site (IRES) (c-kit^CreGFPnls^ or c-kit^Cre^) were crossed with B6.129S6-Gt(ROSA)26Sortm9(CAG-tdTomato)Hze/J (abbreviated as R26^floxed-dTomato^) Cre-reporter mice, which harbour a targeted mutation of the Gt(ROSA)26Sor locus with a loxP-flanked STOP cassette preventing transcription of a CAG promoter-driven red fluorescent protein variant (tdTomato), which is expressed following Cre-mediated recombination. Approximately 20% of total cardiac cells and ~60% of c-kit^pos^ cardiac cells from these mice are dTomato^pos^. Approximately 70% of lineage-committed endothelial/mast cell (CD45^pos^/CD31^pos^c-kit^pos^) cardiac cells are recombined to express dTomato, whereas <10% of the CSC-enriched CD45^neg^CD31^neg^/c-kit^pos^ were recombined to become dTomato^pos^ (**g** representative of *n* = 5 hearts). All data are mean ± SD

Wallner et al.^22^ used a constitutive and TAM-inducible c-kit^Cre^ KI mice^10^ to track the fate of the “c-kit+cells” and did not find any increase in “c-kit+cell-derived CMs” after ISO. Their work was based on the assumption that the c-kit^Cre^KI allele in their mouse models efficiently recombines the marker gene in the c-kit^pos^CSCs. However, we have recently shown that all the c-kit^Cre^ KI alleles so far used^10–12^ are very inefficient in driving recombination of the marker gene in many c-kit+cells, particularly the CSCs^18^. This is so because the efficiency of Cre recombination in these mice is directly proportional to the level of c-kit expression in the cell^15,16^, which is high in mast and endothelial (progenitor) cells but many fold lower in the CSCs^18^. Both constitutive c-kit^CreGFPnls/+^ and TAM-inducible c-kit^mERCremER/+^ (c-kit^MCM/+^) mouse lines have a null c-kit allele and thus they are phenotypically similar to W/+mice^33–35^, show the typical white spotting in the coat (Fig. 4a), have a 50% decrease of c-kit expression^18^, and are incompatible with late fetal/early post-natal life in homozygosis^10,12^. The W-mutation phenotype of the c-kit^Cre^KIs mice is confirmed by the size of a prototypical c-kit-dependent organ, such as the testis, from 2-month-old c-kit^Cre^ mice that shows a significant size reduction (Fig. 4b), which, in part, explains the low fertility of the c-kit^Cre^KI lines. Most relevant, the c-kit haploinsufficiency severely impairs the c-kit^pos^CSCs growth, clonal expansion (Fig. 4c, d), and myogenic differentiation^18^. In addition, in constitutive c-kit^CreGFPnls^ mice, the GFP nuclear expression (GFP^nls^) has a surprisingly low coincidence with c-kit expression. Only ~25% of the c-kit^pos^ BM cells expressed detectable GFP^nls^ (Fig. 4e), while the vast majority of c-kit^pos^ cardiac cells were GFP^nls^ negative by FACS and/or tissue immunohistochemistry (Fig. 4f).

ISO (200 mg/Kg) was injected into 12-week-old male double mutant mice, c-kit^CreGFPnls^: R26^floxed-stop-dTomato/+^ and in control R26^floxed-stop-dTomato/+^ littermates^36^, all implanted with subcutaneous mini-pumps to release BrdU for 28 days. In the Cre-constitutive double transgenic mice, all the cells, which have undergone Cre-dependent recombination (in theory, all cells expressing c-kit) should become dTomato^pos^. However, at baseline before ISO, ~70% of Lineage-committed (CD45^pos^/CD31^pos^) c-kit^pos^ cardiac cells (mainly blood and endothelial lineage-derived cells) are dTomato^pos^ (Fig. 4g). On the other hand, only 10 ± 3% of the Lin^neg^(CD45^neg^/CD31^neg^) c-kit^pos^ cells (which include all the CSCs) are dTomato^pos^ (Fig. 4g). Thus, this system is very inefficient and nor reliable to track the resident CSCs and their progeny^15,16,18^.

**Fig. 5:**
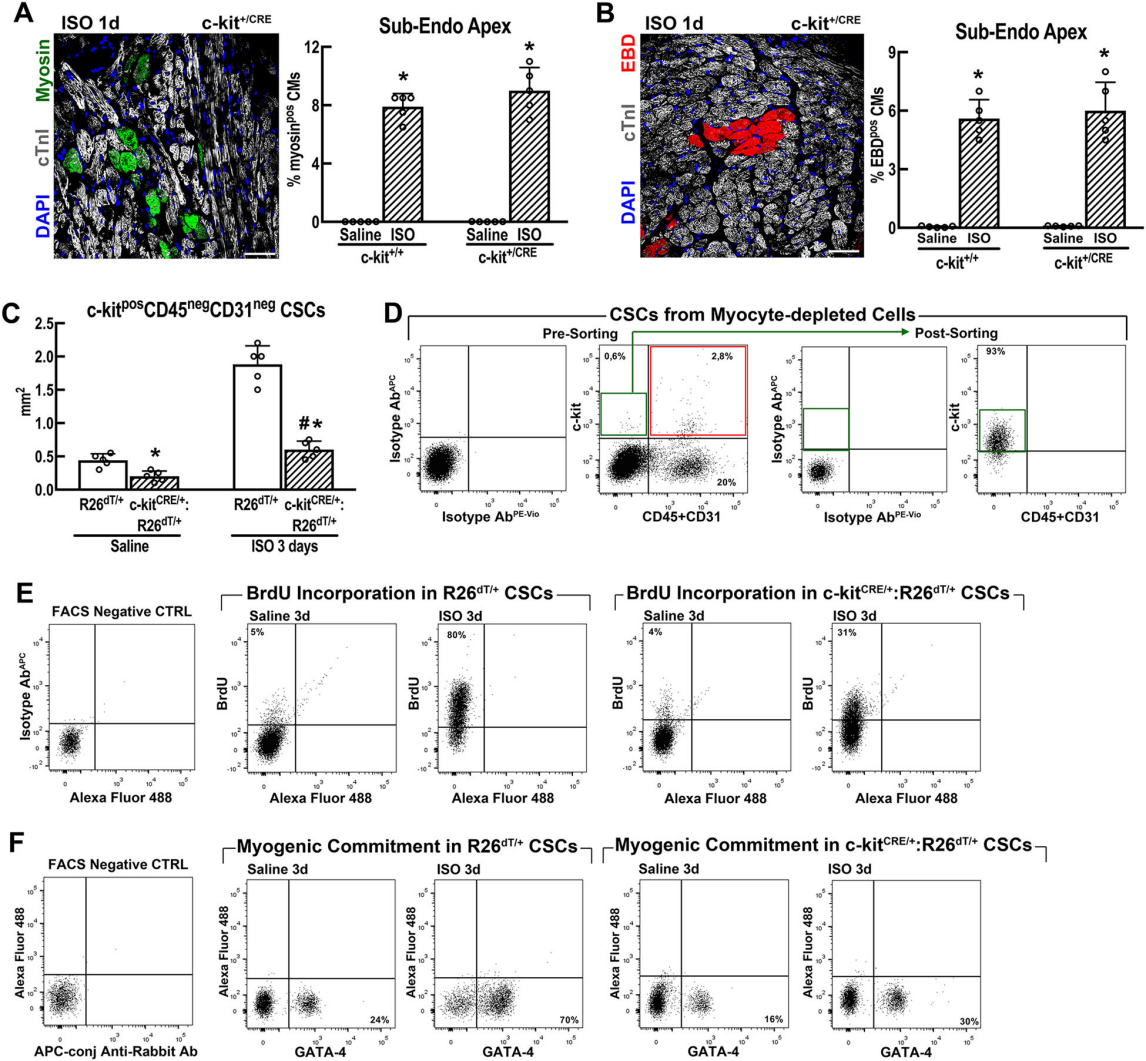
c-kit^Cre^ mice show a defective CSC activation after injury in vivo. **a**, **b** Necrotic cardiomyocytes (CM) (revealed either by myosin Ab in vivo labelling in **a** and EBD incorporation in **b**) were similarly increased in wild-type c-kit^+/+^ and heterozygous c-kit^+/Cre^ mice at 1 day after ISO (200) in the Apex sub-endocardium. *n* = 5 mice per group, *p < 0.05 vs. saline (one-way ANOVA analysis with Tukey’s multiple comparison test). Scale bars = 50 µm. **c** c-kit^CreGFPnls^:R26^floxed-dTomato/+^ (abbreviated as c-kit^Cre/+^:R26^dT/+^) mice show a blunt increase of resident CD45^neg^c-kit^pos^ CSCs when compared with control R26^floxed-dTomato/+^ (abbreviated hereafter as R26^dT/+^) mice 3 days after ISO. *n* = 5 mice per group; *p < 0.05 vs. Saline; ^#^*p* < 0.05 vs. R26^dT/+^ mice (one-way ANOVA analysis with Tukey’s multiple comparison test). **d** Representative FACS detection and isolation of CSCs from cardiomyocyte-depleted cardiac cell preparations. Left, representative gating strategy using isotype antibodies. Pre-sorting, flow cytometry dot plots show that the majority of total c-kit^pos^ cardiac cells are CD45 or CD31 positive (red box), whereas only a minority are CD45 and CD31 negative (green box). Post sorting, flow cytometry dot plots show that CD45^pos^CD31^pos^ (lin^pos^) cells are efficiently removed from cardiac cells by CD45^−^CD31 sorting and CD45^neg^CD31^neg^c-kit^pos^-sorted cells uniformly express low levels of c-kit. **e** Flow cytometry dot plots (representative of *n* = 3) show BrdU incorporation in CD45^neg^ c-kit^pos^ cardiac cells from saline and ISO-treated R26^dT/+^ and c-kit^Cre/+^:R26^dT/+^mice. BrdU—35mg/Kg bid—was intraperitoneally administered in vivo for 3 days in adult mice every 12 h before killing. **f** Flow cytometry dot plots (representative of *n* = 3) show the fraction of myogenic-committed Gata-4^pos^ CD45^neg^c-kit^pos^ CSCs isolated from saline and ISO-treated R26^dT/+^ and c-kit^Cre/+^:R26^dT/+^ mice. All data are mean ± SD

In a separate experiment with c-kit^CreGFPnls/+^ (abbreviated also as c-kit^CRE/+^) mice, c-kit^Cre^ hearts show a similar CM necrosis as the control hearts from wt c-kit^+/+^ littermates at 1 day after ISO (Fig. 5a, b). At 3 days after ISO, control R26^floxed-stop-dTomato/+^ (abbreviated also as R26^dT/+^) hearts show a significant increase in the number of resident CD45^neg^CD31^neg^c-kit^pos^CSCs, whereas the c-kit^CRE/+^:R26^dT/+^ hearts only show a minimal increase when compared with the respective uninjured controls (Fig. 5c). In the uninjured control R26^dT/+^ mice, the majority of freshly isolated CD45^neg^CD31^neg^c-kit^pos/low^CSCs (a representative scheme of their identification and isolation is presented in Fig. 5d) are quiescent with only 5 ± 1% in the cell cycle, as measured by BrdU incorporation (Fig. 5e). In these mice, ISO injury caused a rapid and significant egress of the CSC pool from the quiescent state with 80 ± 8% of activated BrdU^pos^CSCs at 3 days (Fig. 5e). In addition, transient amplifying and myogenic-committed c-kit^pos^Gata-4^pos^ cardiac progenitors significantly increased after ISO compared with vehicle-treated mice (Fig. 5f). In contrast, when compared with control R26^dT/+^ mice, both the percentage of BrdU^pos^CSCs and myogenic-committed c-kit^pos^Gata^pos^ cardiac progenitors were much lower in c-kit^CRE/+^:R26^dT/+^ mice after ISO (Fig. 5e, f).

**Fig. 6:**
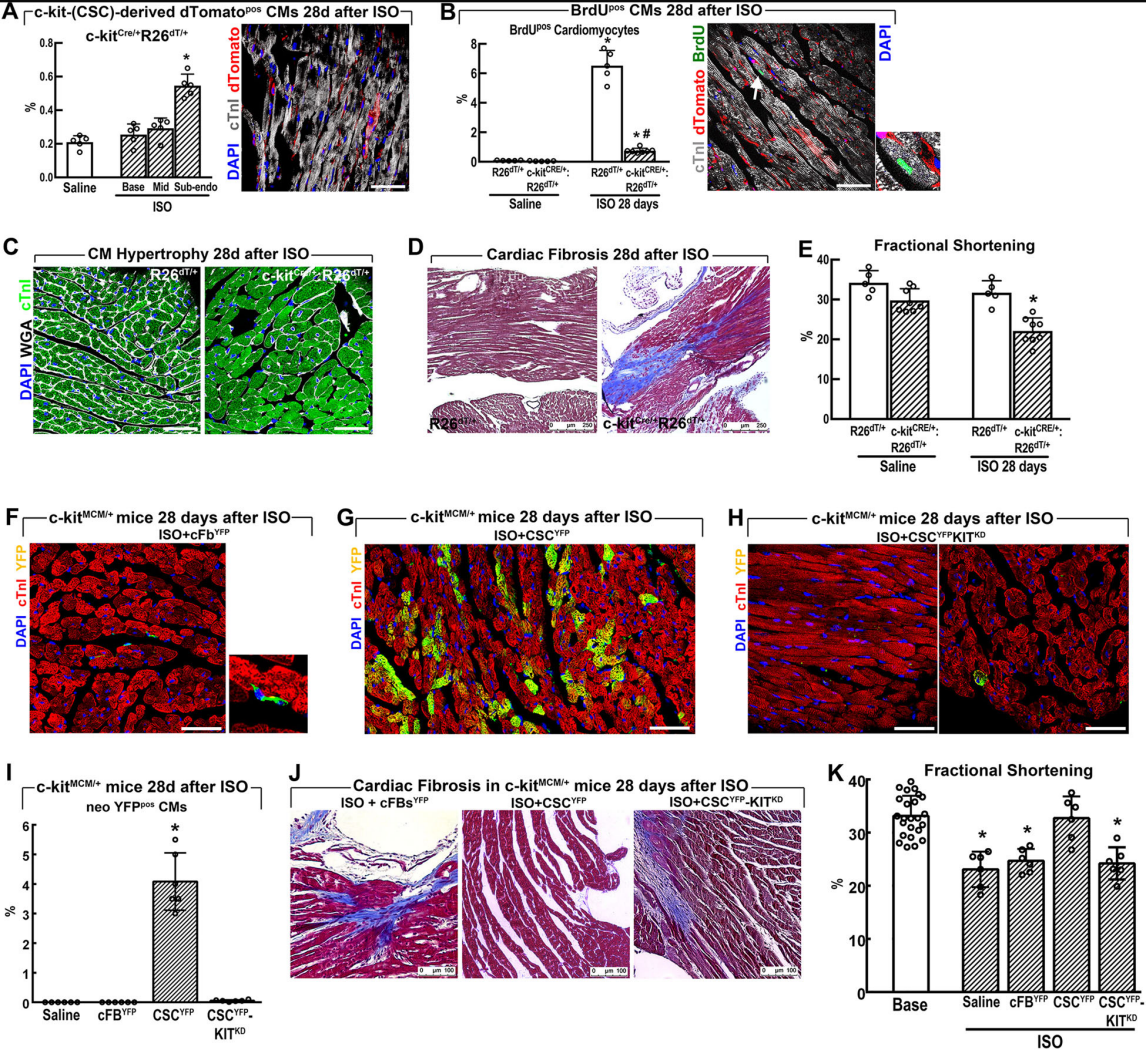
Wild-type c-kit^pos^ CSCs rescue the regenerative defect of c-kit^Cre^ mice after injury in vivo. **a** Bar graph with cumulative data showing the number of dTomato^pos^ myocytes in saline (*n* = 5) and ISO (*n* = 5) injected c-kit^Cre/+^:R26^dT/+^ mice 28 days after treatment. *p < 0.05 vs. all (one-way ANOVA analysis with Tukey’s multiple comparison test). Right, representative confocal image showing dTomato^pos^ CMs in c-kit^Cre/+^:R26^dT/+^ mice. **b** Bar graph with cumulative data shows that 28 days post-ISO, the number of newly generated BrdU^pos^ myocytes was significantly less in c-kit^Cre/+^:R26^dT/+^ (*n* = 7) compared with R26^dT/+^ mice (*n* = 5). *p* < 0,05 vs. Saline; ^#^*p* < 0,05 vs. R26^dT/+^ mice (one-way ANOVA analysis with Tukey’s multiple comparison test). Right, representative confocal image showing BrdU^pos^ CM in c-kit^Cre/+^:R26^dT/+^ mice 28 days after ISO. **c** Representative confocal images of cardiac cross-section showing cardiomyocyte hypertrophy in c-kit^Cre/+^:R26^dT/+^ mice when compared with R26^dT/+^ mice (WGA, wheat germ agglutinin, Cy5 staining, and white fluorescence; cTnI, green; DAPI, blue nuclei). **d** Representative Masson’s trichrome staining of cardiac cross-sections from c-kit^Cre/+^:R26^dT/+^ mice 28 after ISO showing multiple areas of replacement fibrosis compared with R26^dT/+^ mice. Scale bar = 250 μm. **e** Cardiac function 28 days after ISO as assessed by Echocardiography is depressed in c-kit^Cre/+^:R26^dT/+^ (*n* = 8) when compared with R26^dT/+^ mice (*n* = 5). *p < 0.05 vs. all (one-way ANOVA analysis with Tukey’s multiple comparison test). **f** Representative confocal microscopy image showing the absence of YFP^pos^ CMs in cardiac Fibroblasts (cFb^YFP^)-injected c-kit^MCM/+^ mice (right insert, 3× zoom, shows the rare detection of YFP^pos^ interstitial non-CM cells) in the apical sub-endocardium 28 days after ISO injury. Scale bar = 50 µm. **g** Robust replacement of YFP^pos^ CMs in CSC^YFP^-injected c-kit^MCM/+^ mice in the apical sub-endocardium 28 days after ISO injury. Scale bar = 50 µm. **h** The two representative confocal images show the very minimal number of YFP^pos^ CMs (only visible in the right panel) in CSC^YFP^-KIT^KD^(c-kit knock-down by specific stealth RNAi siRNA transfection)-injected c-kit^MCM/+^ mice in the apical sub-endocardium 28 days after ISO injury where most of the engrafted cells remained as interstitial non-CM cells. Scale bar = 50 µm. **i** Bar graph with cumulative data showing the number of YFP^pos^ cardiomyocytes (CMs) in injured c-kit^MCM/+^ mice injected with saline (*n* = 5), CSC^YFP^ (*n* = 5), or CSC^YFP^-KIT^KD^ (*n* = 5) injected c-kit^Cre/+^:R26^dT/+^ mice 28 days after ISO. *p < 0.05 vs. all (one-way ANOVA analysis with Tukey’s multiple comparison test). **j** Representative Masson’s Trichrome staining showing the presence of cardiac fibrosis in c-kit^MCM/+^ mice 28 days after ISO injected with cFBs^YFP^ or CSC^YFP^-KIT^KD^ that is absent in c-kit^MCM/+^ mice transplanted with CSC^YFP^. Scale bar = 100 μm. **k** Cumulative data showing Fractional Shortening as assessed by Echocardiography in c-kit^MCM/+^ mice at baseline and 28 days after ISO plus Saline or cFBs^YFP^ or CSC^YFP^ CSC^YFP^-KIT^KD^. *n* = 6 per group, *p* < 0.05 vs. Base and CSC^YFP^ (one-way ANOVA analysis with Tukey’s multiple comparison test). Scale bars = 50 µm unless differently specified in the panels. All data are mean ± SD

At 28 days after ISO, we detected only a slight increase of c-kit^pos^ cell-derived dTomato^pos^CMs (0.55 ± 0.07% vs. 0.21 ± 0.04%, *p* < 0.05) in the sub-endocardial apex of ISO-injured vs. saline-injected c-kit^CRE/+^:R26^dT/+^ mice (Fig. 6a). The number of BrdU^pos^ CMs was also significantly lower in the c-kit^CRE/+^:R26^dT/+^ mice than in the R26^dT/+^ control littermates (0.75 ± 0.17% vs. 6.52 ± 1.04%, *p* < 0.05) (Fig. 6b). The severe deficit of CM regeneration in c-kit^CRE/+^:R26^dT/+^ mice was accompanied by hypertrophy of the surviving pre-existing CMs, which was absent in R26^dT/+^control mice (Fig. 6c). Also, although the hearts of R26^dT/+^ control mice at 28 days after ISO did not show evidence of fibrosis, there was multiple areas of fibrosis in the sub-endocardial apex of the c-kit^CRE/+^:R26^dT/+^ mice, in agreement with the decreased CM replacement (Fig. 6d). Twenty-eight days after ISO, the lack of robust CM regeneration was accompanied by persistence of LV functional impairment in c-kit^CRE/+^:R26^dT/+^, which was absent in R26^dT/+^ control mice (Fig. 6e).

### c-kit hemizygosity supresses CSC myogenicity and regenerative potential

We tested whether transplantation of exogenous wtCSCs would rescue the defective cardioregenerative phenotype of c-kit^Cre^KI mice. To this end, c-kit^mERCremER/+^ mice (hereafter c-kit^MCM/+^ mice)^10^ were ISO-injured (200 mg/Kg). Twelve hours later, they were administered through the tail vein with saline or 4 × 10^5^ each of either wt YFP^+^ CSCs (CSC^YFP^) or wt YFP^+^ cFBs (cFBs^YFP^) (Fig. 6f, g). To further assess c-kit gene function in CSC regenerative potential in vivo, additional ISO-injured mice were similarly transplanted with CSC^YFP^ transfected with a *KIT*^*siRNA*^ knocking down c-kit expression (Supplementary Fig. 4). At 28 days after ISO, the transplanted exogenous wtCSC^YFP^ had differentiated into new YFP^+^ CMs in the sub-endocardial layer where they reached 4 ± 1% of total CMs (Fig. 6g, i), which were clearly absent in cFBs transplanted mice (Fig. 6f, i). c-kit knockdown significantly reduced wtCSC myogenic potential, as indeed CSC^YFP^-KIT^KD^ did not generate significant new CMs (Fig. 6h, i). New CM formation, derived from the transplanted wtCSC^YFP^, prevented cardiac fibrosis when compared with the recipients of either cFBs^YFP^, CSC^YFP^-KIT^KD^, or saline (Fig. 6j). wt CSC^YFP^ transplantation in c-kit^MCM/+^ mice also normalized cardiac function, whereas the saline-, cFBs-, and CSC^YFP^-KIT^KD^-injected mice remained in HF (Fig. 6k).

### CSC activation and ensuing CM formation depends on a proper level of c-kit function

To devoid the hearts of functional resident CSCs, ISO injury was followed by 5-FU treatment^21^. This model invariably leads to HF in rats and mice^21^. 5-FU regime ablated proliferating CSCs and resulted in a severe CSC deficit, absent CM replacement, continued CM loss, hypertrophy of surviving CMs and dilated decompensated cardiomyopathy (Supplementary Fig. 5A–F). The lack of CSC-derived CM regeneration in ISO + 5-FU cardiomyopathy was confirmed using double transgenic Tg-myh6^MCM^:R26^mT/mG^ mice after TAM treatment (Supplementary Fig. 5G).

**Fig. 7:**
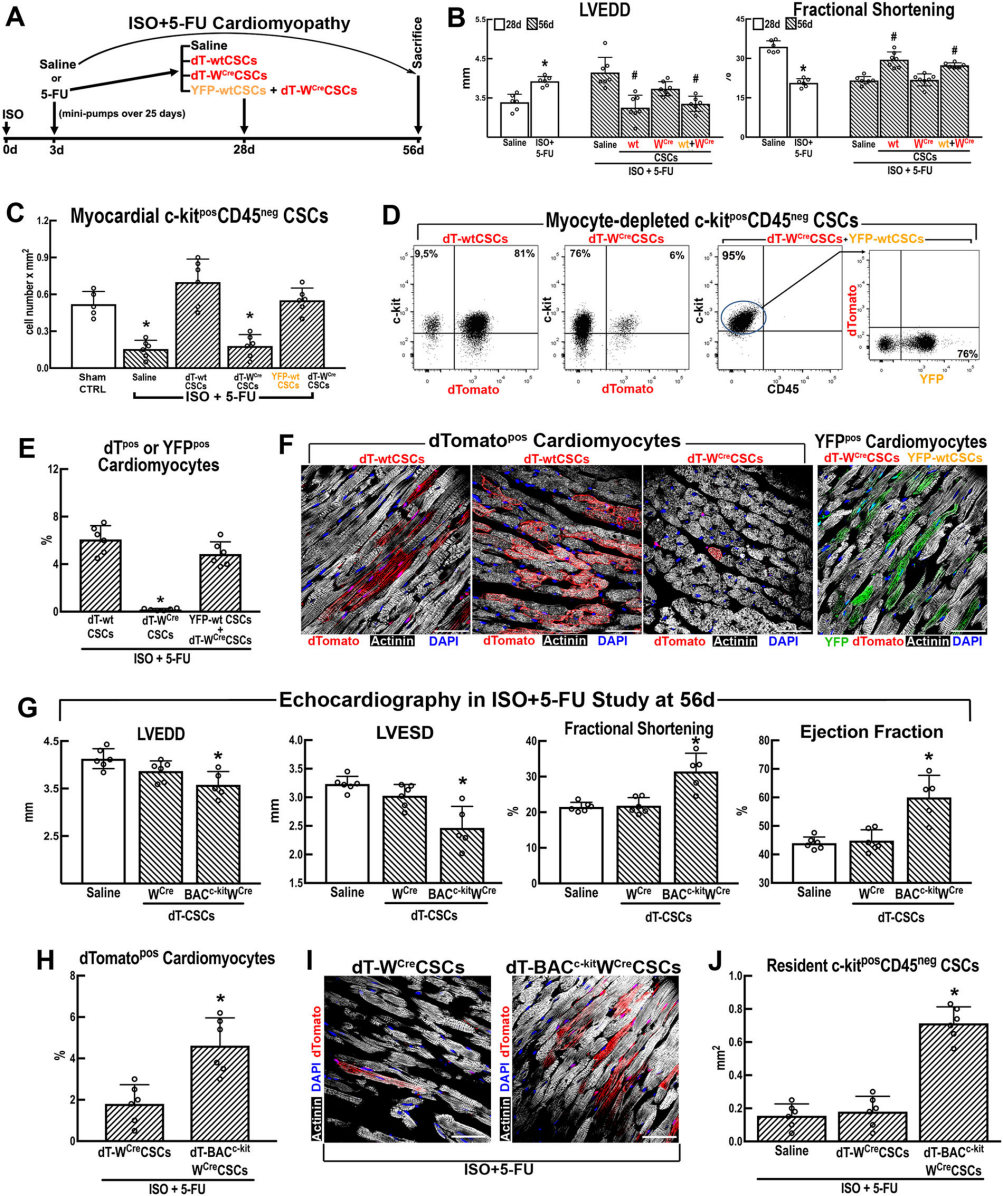
c-kit haploinsufficiency impairs the regenerative potential of c-kit^Cre/+^ CSCs in vivo. **a** Schematic of ISO + 5-FU cardiomyopathy study design. **b** LVEDD and LV Fractional Shortening representative images by Echo in male wild-type C57BL/6J mice 28 days after saline or ISO + 5-FU (white bars) and 56 days after ISO + 5-FU administration treated with tail vein injections of saline (*n* = 6), dT-wtCSCs (*n* = 7), dT-W^Cre^CSCs (*n* = 7), or YFP-wtCSCs+dT-W^Cre^CSCs (*n* = 6). *p < 0.05 vs. Saline and #*p* < 0.05 vs. ISO + 5-FU (one-way ANOVA analysis with Tukey’s multiple comparison test). **c** Myocardial ckit^pos^CD45^neg^CSC number in the different groups of animals as above. *p < 0.05 vs. all (except*) (one-way ANOVA analysis with Tukey’s multiple comparison test). *n* = 5 for sham control (CTRL) and *n* = 6 for ISO + 5-FU groups (one-way ANOVA analysis with Tukey’s multiple comparison test). **d** Flow cytometric analysis of dTomato^pos^ (or YFP^pos^) cell fraction within the c-kit^pos^CD45^neg^ CSC compartment 28 days after CSC injections in mice with ISO + 5-FU cardiomyopathy. *N* = 8 per group. **e** Bar graph showing cumulative data of CSC-derived Tomato^pos^ or YFP^pos^ CMs in mice with ISO + 5-FU cardiomyopathy. *p < 0.05 vs. all (*n* = 6) (one-way ANOVA analysis with Tukey’s multiple comparison test). **f** Confocal microscopy representative images of CSC-derived newly formed dTomato^pos^ (or YFP^pos^) CMs after the respective CSC injection in mice with ISO + 5-FU cardiomyopathy (scale bar 50 μm). In dT-wtCSC-treated hearts, CSC-derived newly formed dT(Tomato)^pos^ CMs were several times detected as spots of apparent clonal amplification (mid panel). **g** Echocardiographic data and LV images at 56 days after ISO + 5-FU treatment and 28 days after either saline (*n* = 6), W^Cre^CSCs (*n* = 6), or BAC^c-kit^W^Cre^CSCs (*n* = 5) injection; *p < 0.05 vs. all (one-way ANOVA analysis with Tukey’s multiple comparison test). **h**, **i** Percentage of CSC-derived dTomato^pos^ CMs in ISO + 5-FU cardiomyopathy (left); *p < 0.05 vs. W^Cre^CSCs (*n* = 6) (one-way ANOVA analysis with Tukey’s multiple comparison test); (right) confocal microscopy representative images of CSC-derived newly formed dTomato^pos^ CMs after the respective CSC injection in ISO + 5-FU cardiomyopathy. **j** Myocardial c-kit^pos^CD45^neg^CD31^neg^CSC number in the different groups of animals with ISO + 5-FU cardiomyopathy. *p < 0.05 vs. all (*n* = 6) (one-way ANOVA analysis with Tukey’s multiple comparison test). Scale bars = 50 μm. All data are mean ± SD

Next, we addressed whether the CSCs are sufficient to regenerate the cardiac tissue lost and to restore myocardial function after ISO injury. If so, whether the CSC regenerative properties are dependent on a normal diploid c-kit level. We compared in vivo the regenerative properties of cloned W^Cre^CSCs (so called for the W phenotype of the c-kit^CRE^ mice) obtained from c-kit^CreER(T2)/+^:R26^mT-mG/+^ mice (hereafter dT-W^Cre^ CSCs) with cloned wtCSCs from R26^mT-mG/+^ littermates (dT-wtCSCs). A total of 4 × 10^5^ cloned cells of each type (all of them the progeny of a single cell) were transplanted through the systemic circulation by tail vein injection, into 16-week-old syngeneic male C57BL/6J mice with ISO + 5-FU failing cardiomyopathy^21^ (Fig. 7a). Control ISO + 5-FU mice were injected an equal volume of saline. One month after treatment, all saline-injected mice and those transplanted with dT-W^Cre^CSCs were still in overt cardiac failure (Fig. 7b, Supplementary Fig. 6A). In contrast, those treated with dT-wtCSCs had completely recovered from cardiac dysfunction (Fig. 7b, Supplementary Fig. 6A). In addition, although the dT-wtCSCs reconstituted the host’s myocardial c-kit^pos^CSC pool previously ablated by the ISO + 5-FU regime, the dT-W^Cre^CSCs did not and the myocardium of the relative recipient animals was practically devoid of CSCs (Fig. 7c, d). Concordantly, dTomato^pos^ newly formed CMs (6.08 ± 1.16%) were detected in the mice injected with dT-wtCSCs (Fig. 7e), whereas the dT-W^Cre^CSCs minimally contributed dTomato^pos^ CMs (0.18 ± 0.08%) in the ISO + 5-FU failing hearts (Fig. 7e). None of these effects was due to differential tissue-homing and engraftment of dT-wtCSCs and dT-W^Cre^CSCs, as similar fractional numbers of each were detected in the myocardium 24 h after cell transplantation (Supplementary Fig. 6B).

The regenerative phenotype of W^Cre^CSCs was further tested in a competitive reconstitution assay by co-injecting YFP-positive (YFP^pos^) wtCSCs (obtained from R26^stopYFP^ reporter mice^21^) and dT-W^Cre^CSCs in a 1:3 ratio (a total of 4 × 10^5^ cells injected) into mice with ISO+5-FU cardiomyopathy. At 28 days, the YFP^pos^wtCSC+W^Cre^CSC combination had partially reverted the cardiac dysfunction and CSC pool in all the animals treated (Fig. 7a–c and Supplementary Fig. 6A). However, in these hearts—although the YFP^pos^wtCSC were only 25% of the CSCs transplanted—the Lin^neg^c-kit^pos^CSC pool was constituted exclusively by YFP^pos^wtCSCs with no detectable persistence of dT-W^Cre^CSCs (Fig. 7d). Also, the newly formed CMs were exclusively YFP^pos^ (4.85 ± 1.03%) with only an occasional dTomato^pos^ CM (< 0.01%) (Fig. 7e, f).

To test whether restoring the diploid level of c-kit expression in the dT-W^Cre^CSCs would correct their myogenic and regenerative defects in vivo, we transfected a BAC construct spanning the entire c-kit gene locus^25^ into cloned dT-W^Cre^CSCs, as recently reported^18^. This clone carries a single BAC/c-kit copy (hereafter dT-BAC^c-kit^W^Cre^CSCs) and expresses c-kit mRNA and protein levels similar to those of dT-wtCSCs and doubled those of the untransfected parent c-kit/haploinsufficient dT-W^Cre^CSCs^18^. Adult C57BL/6J male mice with ISO + 5-FU-induced cardiomyopathy were injected as above with either dT-BAC^c-kit^W^Cre^CSCs, unedited BAC-naïve dT-W^Cre^CSCs, or saline. At 56 days, mice injected with dT-W^Cre^CSCs remained in overt HF similar to saline-injected mice (Fig. 7g). In striking contrast and comparable to dT-wtCSCs (Fig. 7b and Supplementary Fig. 6A), the dT-BAC^c-kit^W^Cre^CSCs (*n* = 5) had fully restored cardiac function (Fig. 7g). The dT-BAC^c-kit^W^Cre^CSCs produced new dTomato^pos^ CMs (~5%) in an amount similar to the dT-wtCSCs (Fig. 7h, i). In addition, dT-BAC^c-kit^W^Cre^CSCs had restored the resident CSC cohort in the host myocardium (Fig. 7j).

Lastly, dT-wtCSCs from R26^mT/mG^ mice were injected into Tg-myh6^MCM^ mice with ISO + 5-FU cardiomyopathy, fed with a TAM diet for 28 days. In this genetic arrangement, all dTomato^pos^ CMs (6.5 ± 2%) were invariably only dTomato^pos^, with none of them co-expressing GFP (Supplementary Fig. 7). These data rule out cell fusion as a relevant mechanism for new CMs contributed by the c-kit^pos^CSCs.

## Discussion

The data presented here documents that CSCs from the c-kit^Cre^ (also named as W^Cre^) KI mice are impaired in their myogenic and cardiac regenerative properties because of their c-kit hemizygosity. These defects are fully rescued by BAC transgenesis of a single c-kit copy into the c-kit^Cre^CSCs to restore c-kit diploidy. On the other hand, worsening cardiac remodelling after injury in c-kit^Cre^ mice is reversed by the transplantation of wt c-kit^+/+^CSCs. Therefore, the CSC’s myogenic and regenerative properties are c-kit dose dependent^18^ and require a diploid level of c-kit expression. For this reason, all the c-kit^Cre^ KI mouse lines, which abolish c-kit expression from the c-kit^Cre^KI allele^10–12^, cannot and should not be used to identify or to track the fate of the CSCs either in vitro or in vivo.

Acute excess catecholamines directly damage the rodent/murine heart^37–41^ with resultant CM death^37–41^. Using ISO overdose, together with different genetic rodent models, we had previously shown that the CSCs are the main adult myocardium regenerative agents, which are necessary and sufficient for myocardial repair/regeneration after damage^21^. Houser and colleagues^42^ also reported that repair of the ISO-injured adult heart involves the generation of new CMs derived from resident progenitors^42^. This same group, however, using wild-type and c-kit^Cre^KI mouse models^22^, has recently reported to find no evidence of CM death or any increased CM formation after ISO, raising questions about the ISO-damage model and the role of the CSCs^22^.

The present study confirms and extends our previous results^21^, and reconciles them with those of Wallner et al.^22^. The main reasons for the discordance with Wallner et al.^22^ are the different myocardial areas sampled (entire LV^22^ vs. sub-endocardial apex^21^) and the type, dose, and duration of the nucleoside analogue used to detect new cell formation. Considering cumulative EdU dose, Wallner et al.^22^ under-labelled the replicating cells by about tenfold when compared with our experimental design^21^. The discrepancy in the results agrees with the well-known fact that efficiency of EdU and BrdU labelling of dividing cells is proportional to the cumulative dose used^43^.

The major pitfall of the reported null CSC contribution to new CMs after ISO in Wallner et al.^22^ however is due to the use of a c-kit^cre^KI genetic mouse line^10^. The ongoing CSC controversy has been based on two assumptions: (a) that all the myocardial c-kit^pos^ cells are expected to behave like *bona fide* CSCs^10^, whereas in reality they are only ~1–2% of the c-kit^pos^ cells;^17,18^ (b) that the genetic tagging system used^10–12^ does in fact tags the majority of the true c-kit^pos^CSCs. As we have recently shown, also the latter is incorrect^18^. All mouse lines with Cre KIs into the c-kit locus^10–12^ have rendered the targeted allele a null mutant with concomitant c-kit receptor protein deficiency, which is equivalent to the W mutation^44,45^. Because of the low level of c-kit expression in most stem cell types^44,46^ and particularly in the c-kit^pos^CSCs, which have the lowest level so far detected^18^, the hemizygous KI Cre allele fails to produce enough Cre to induce recombination of the marker gene in sufficient number of c-kit^pos^CSCs to identify them, track their fate, and, more importantly, their progeny^18^.

At steady state, ~95% of the CSCs in the adult mammalian heart are quiescent, whereas 3–5% are cycling and differentiating^21^. As shown here (Fig. 5) and elsewhere^20,21^, in rapid response to ISO damage, most CSCs (~80%) in wt animals are rapidly activated, re-enter the cell cycle, multiply three- to fourfold and commit to differentiate with ample production of CMs to replace those lost to ISO. In contrast, all c-kit hypomorphs—W mutants and c-kit^Cre^KI mouse lines—show serious growth and differentiation defects in different stem and somatic cell types^44,45^, including the CSCs^18^. The c-kit^Cre^CSCs have lost their myogenic potential both in vivo (Fig. 6 and 7) and in vitro^18^. These findings point towards a required minimal level of c-kit protein for myogenicity, regenerative potential, and CSC niche replacement.

Based on the data presented here, this c-kit requirement could be cell-intrinsic, extrinsic—due to effects of the mutation on the niche or others on the myocardial properties—or to a combination of both. The ISO + 5-FU-induced dilated failing cardiomyopathy of wild-type animals can be rescued by transplantation of clone-derived exogenous wtCSCs, which exhibit robust myogenic potential but not by transplantation of W^Cre/+^CSCs (Fig. 7). This result, together with the in vivo competition assay between wtCSCs and the transplantation of W^Cre^CSCs into wt ISO + 5-FU animals (Fig. 7) clearly demonstrate that, although both cell types show similar homing to the damaged myocardium when injected through the systemic circulation, the mutant CSCs phenotype is not corrected in the wt myocardium, which suggests that the defect is cell-intrinsic. This hypothesis is confirmed by the correction of the mutant phenotype and restauration of the in vivo regenerative properties of the W^Cre/+^ mutant CSCs by transgenesis of a single-copy of the c-kit^wt^ locus, which restores their c-kit diploid state (Fig. 7). This result is consistent with the restoration of all the in vitro parameters tested^18^, which together convincingly show that c-kit-expressing CSCs are capable to regenerate the main cellular components of the adult myocardium for which a diploid level of c-kit receptor function is required.

## Supplementary information

Supplementary Figures and Tables
