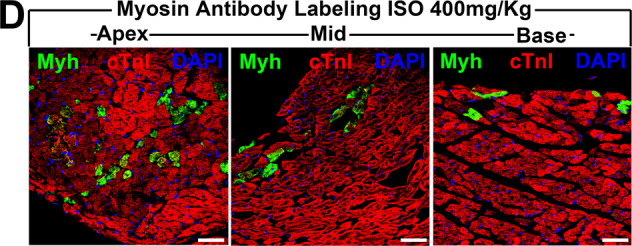# Correction: c-kit Haploinsufficiency impairs adult cardiac stem cell growth, myogenicity and myocardial regeneration

**DOI:** 10.1038/s41419-023-05798-w

**Published:** 2023-04-12

**Authors:** Iolanda Aquila, Eleonora Cianflone, Mariangela Scalise, Fabiola Marino, Teresa Mancuso, Andrea Filardo, Andrew J. Smith, Donato Cappetta, Antonella De Angelis, Konrad Urbanek, Andrea M. Isidori, Michele Torella, Valter Agosti, Giuseppe Viglietto, Bernardo Nadal-Ginard, Georgina M. Ellison-Hughes, Daniele Torella

**Affiliations:** 1 Molecular and Cellular Cardiology, Department of Experimental and Clinical Medicine, Magna Graecia University, Catanzaro, 88100 Italy; 2 Faculty of Biological Sciences, School of Biomedical Sciences, University of Leeds, Leeds, LS2 9JT UK; 3 Section of Pharmacology, Department of Experimental Medicine, University of Campania “L.Vanvitelli”, Naples, 80121 Italy; 4 Department of Experimental Medicine, “La Sapienza” University, Rome, 00161 Italy; 5 Department of Cardiothoracic Surgery, University of Campania “L.Vanvitelli”, Naples, 80121 Italy; 6 Interdepartmental Center of Services (CIS) and Department of Experimental and Clinical Medicine, University Magna Graecia, Campus Salvatore Venuta-Viale Europa, Catanzaro, 88100 Italy; 7 Faculty of Life Sciences, School of Basic and Medical Biosciences, King’s College London, Guy’s Campus, London, SE1 1UL UK

Correction to: *Cell Death and Disease* 10.1038/s41419-019-1655-5, published online 04 June 2019

The original version of this article contained an error in figure 1. For a mere and genuine human mistake, the MID and BASE representative images in Fig. 1D were inadvertently duplicated during the assembling of the multiple panels of figure 1, and the actual BASE image for Figure 1D was not shown. The correct Figure 1D, including the actual Base image, is now available with this correction. The authors sincerely apologize for the error.