# Supplementary material for: c-kit Haploinsufficiency impairs adult cardiac stem cell growth, myogenicity and myocardial regeneration
Source: Cell Death Dis. 2019 Jun 4;10(6):436. doi: 10.1038/s41419-019-1655-5 (PMC6547756; doi:10.1038/s41419-019-1655-5)
Supplement: Supplementary file 1 — Supplementary Figures and Tables [file 41419_2019_1655_MOESM1_ESM.pdf]

SUPPLEMENTARY MATERIAL

c-kit Haploinsufficiency Impairs Adult Cardiac Stem Cell Growth, Myogenicity and Myocardial Regeneration

Iolanda Aquila[1*], Eleonora Cianflone[1*], Mariangela Scalise[1], Fabiola Marino[1], Teresa Mancuso[1], Andrea Filardo[1], Andrew J. Smith[1,2], Donato Cappetta[3], Antonella De Angelis[3], Konrad Urbanek[3], Andrea M. Isidori[4], Michele Torella[5], Valter Agosti[6], Giuseppe Viglietto[6], Bernardo Nadal-Ginard[1#], Georgina M. Ellison-Hughes[6#], Daniele Torella[1#]

* These two authors equally contributed to this study; # These authors share senior authorship.

Running Title: c-kit Haploinsufficiency Impairs Adult Cardiomyogenesis

Affiliations: [1]Molecular and Cellular Cardiology, Department of Experimental and Clinical Medicine, Magna Graecia University, Catanzaro, 88100, Italy;

[2]School of Biomedical Sciences, Faculty of Biological Sciences, University of Leeds, Leeds, UK, LS2 9JT;

[3]Department of Experimental Medicine, Section of Pharmacology, University of Campania “L.Vanvitelli”, Naples, 80121, Italy

[4]Department of Experimental Medicine, “La Sapienza” University, Rome, 00161, Italy;

[5]Department of Cardiothoracic Surgery, University of Campania “L.Vanvitelli”, Naples, 80121, Italy;

[6]Interdepartmental Center of Services (CIS) and Department of Experimental and Clinical Medicine, University Magna Graecia, Catanzaro, Campus Salvatore Venuta - Viale Europa, 88100, Catanzaro, Italy.

[7]School of Basic and Medical Biosciences, Faculty of Life Sciences &Medicine, Kings College London, Guy's campus, London SE1 1UL, UK.

SUPPLEMENTARY FIGURE 1

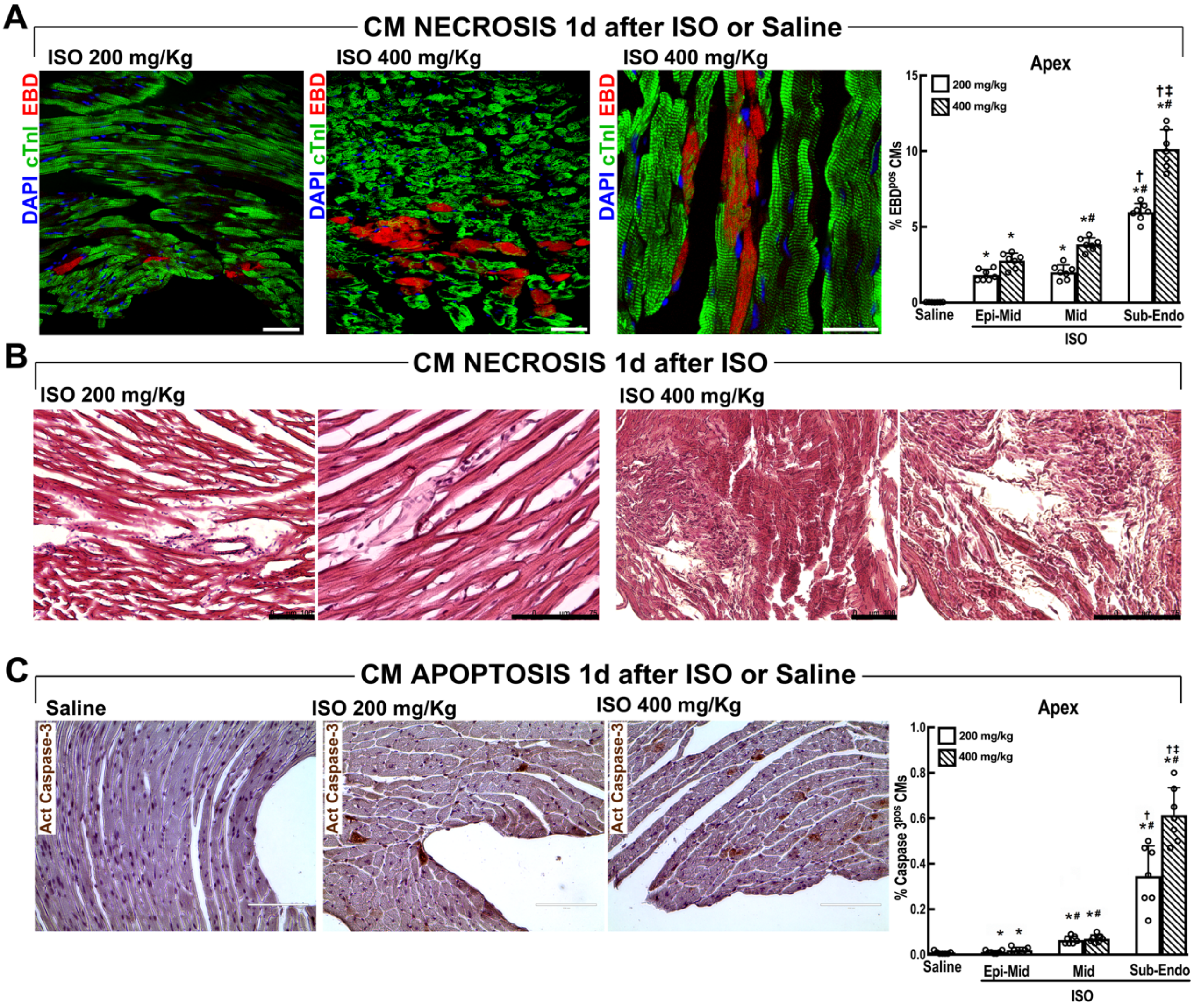

Suppl. Fig. 1. Cardiomyocyte Death by acute excessive catecholamines. A, Representative confocal microscopy images showing Evans Blue Dye (EBD) *in vivo* incorporation 1 day after ISO 200 or 400 mg/Kg within the LV Apex. Right, cumulative data showing number of necrotic EBD^{pos} CMs after ISO 200 (open boxes) or 400 mg/Kg (black boxes) in Epicardium (Epi-Mid), Myocardium (Mid) and Sub-Endocardium (Sub-Endo). (*n*=7 per group) *p<0.05 vs. saline; #p<0.05 vs. Epi; †p<0.05 vs. Mid; ‡p<0.05 vs. 200 mg/Kg) (One-way ANOVA analysis with Tukey's multiple comparison test). Scale bar=50μm. **B,** Representative hematoxylin and eosin (H&E) cross sections of the apical LV endocardium at 40x and 60x magnifications 1 day after ISO 200 or 400 mg/Kg. Typical features of necrotic death are evident. Scale bar=75 μm (in lower magnification left and mid-right panels) and 100 μm (in higher magnification mid-left and right panels).
C, Representative pictures of apoptotic CMs stained by activated caspase 3 (DAB staining, brown) 1 day after ISO 200 or 400 mg/Kg. Apoptotic (caspase-3 positive) CMs within the LV Apex were significantly increased in a dose dependent manner at 1 day after ISO with higher frequency in sub-endocardium. (*n*=7 per group) *p<0.05 vs. saline; #p<0.05 vs. Epi; †p<0.05 vs. Mid; ‡p<0.05 vs. 200 mg/Kg) (One-way ANOVA analysis with Tukey's multiple comparison test). Scale bar=100 μm.
All data are Mean ± SD.

SUPPLEMENTARY FIGURE 2

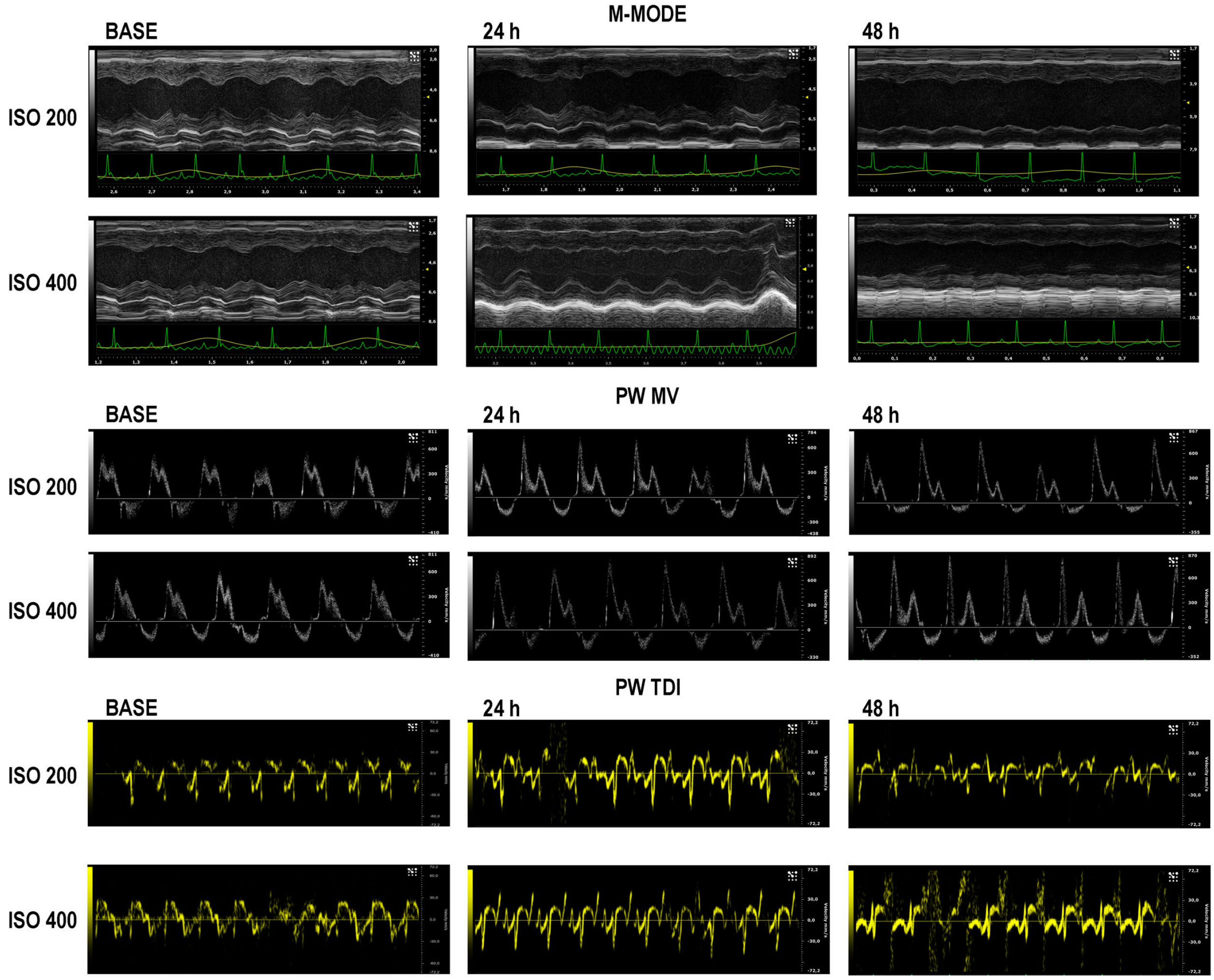

Suppl. Fig. 2. Isoproterenol transiently impairs cardiac function. Uncropped Echo images from main Figure 2 showing scale bars.

SUPPLEMENTARY FIGURE 3

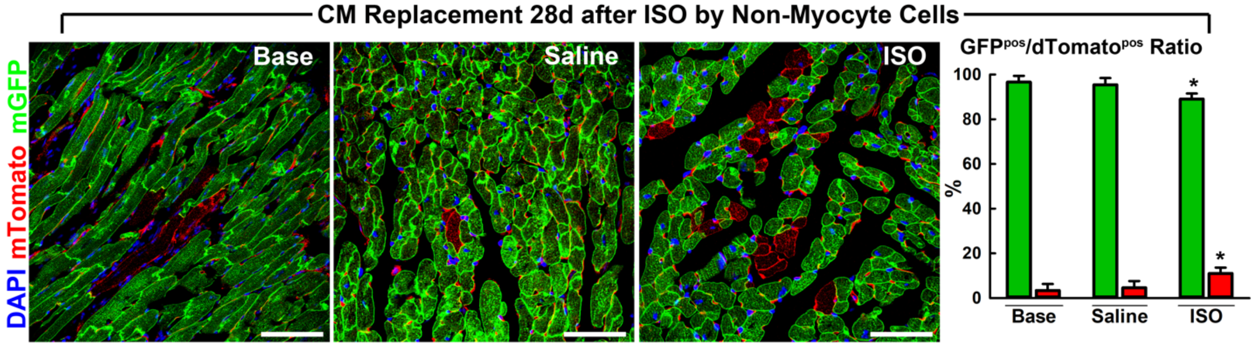

Suppl. Fig. 3. Cardiomyocyte Replenishment after ISO does not orginate from pre-existing cardiomyocyte division. Representative confocal microscopy images of GFP and dTomato fluorescence in the sub-endocardial, apical layer of Tg-myh6^{MCM}:R26$^{mT/mG}$ mice 28 days after Saline or ISO. Scale bar=50 µm. Right, fraction of GFP^{pos} and dTomato^{pos} CMs in Tg-myh6^{MCM}:R26$^{mT/mG}$ mice 28 days after Saline (*n*=6) and ISO (*n*=6). Base (*n*=7). *$p<0.05$ vs. all. (One-way ANOVA analysis with Tukey's multiple comparison test)* Tg-myh6^{MCM}:R26$^{mT/mG}$ mice were pre-treated with Tamoxifen (40mg/Kg i.p. every other day for 14 days).
All data are Mean ± SD.

<u>Expanded Test for Suppl. Fig. 3:</u>

To further confirm that the BrdU/EdU-labelled CMs after the ISO insult are newly formed and not a subpopulation of spared CMs that have re-entered the cell cycle, we analysed new CM formation using double transgenic myh6-mER-Cre-mER//R26^{mT-mG} mice (abbreviated as Tg-myh6^{MCM}:R26^{mT-mG} mice). These double-mutant mice carry a tamoxifen (TAM)-inducible Cre Recombinase driven by the cardiac α-myosin heavy chain (α-MHC or myh6) promoter and a double fluorescent (dT/mGFP) gene reporter in the ROSA26 locus where, upon Cre-induced recombination, a membrane-bound dimeric tomato (dT) is replaced by a membrane-bound green fluorescent protein (mGFP). Eight-week-old double mutant male mice (*n*=6) were injected in alternate days with TAM (40 mg/Kg i.p.) for 2 weeks and analysed two weeks later (to minimize potential late TAM effects and acute Cre toxicity), at which time 97±2% of their CMs were correctly recombined to express mGFP instead of dTomato (Supplementary Figure 3, Base). No mGFP expression was detected in Tg-myh6^{MCM}:R26^{mT-mG} mice which did not receive TAM where all CMs were uniformly dTomato positive. In TAM-treated mice mGFP and dTomato labelling was mutually exclusive, with only a negligible fraction of CMs double positive for mGFP and dTomato (<0.01%), which likely can be explained by the slow decay of dTomato after Cre-induced recombination. Additional TAM-treated and GFP recombined Tg-myh6^{MCM}:R26^{mT-mG} mice were treated with ISO at 200 mg/Kg or with Saline. At 28 days after ISO these mice had an increase in dTomato^{pos} CMs mainly in the sub-endocardial apex where they reached 10±2% compared to only 4±1% in the same region of Saline-injected control Tg-myh6^{MCM}:R26^{mT-mG} mice (Supplementary Figure 3). No double positive dTomato+mGFP CMs were detected after ISO, excluding that non-CM cells (dTomato^{pos}) could have fused to pre-existing CMs (mGFP^{pos}) to generate cells which could be confused with new CMs. Overall, the increased number of dTomato^{pos} CMs with the concomitant dilution of the mGFP^{pos} CMs after ISO shows that the new CM formation was not due to division of pre-existing CMs (which by nature of the experimental design would have not changed the ratio of dTomato^{pos}/mGFP^{pos} CMs). Also, these data indicate that the new dTomato^{pos} CMs had to have arisen from non-myocyte progenitor/precursor cells which did not express α-MHC during the TAM treatment and, therefore, did not recombine the dTomato/mGFP marker.

SUPPLEMENTARY FIGURE 4

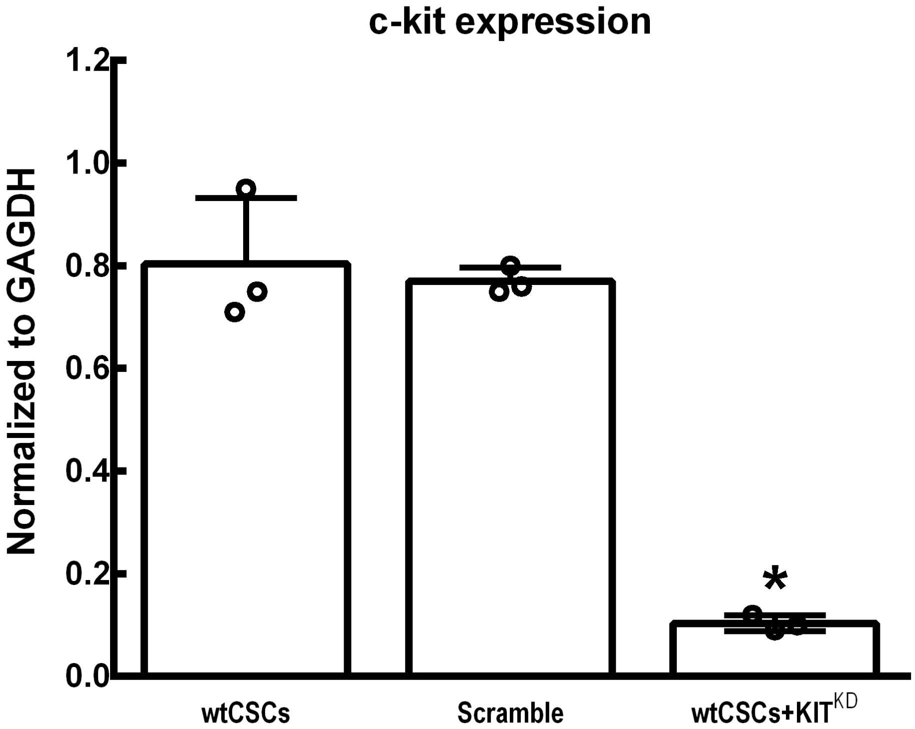

Suppl. Fig. 4. C-kit gene silencing through the transfection of shRNA. RT-PCR data showing the efficient silencing of KIT in wild type CSCs (CSC^{YFP}) using Stealth RNAi KIT^{siRNA} . Scramble refers to Stealth RNAi negative control siRNA transfection. *$p<0.05$ vs. all.

SUPPLEMENTARY FIGURE 5

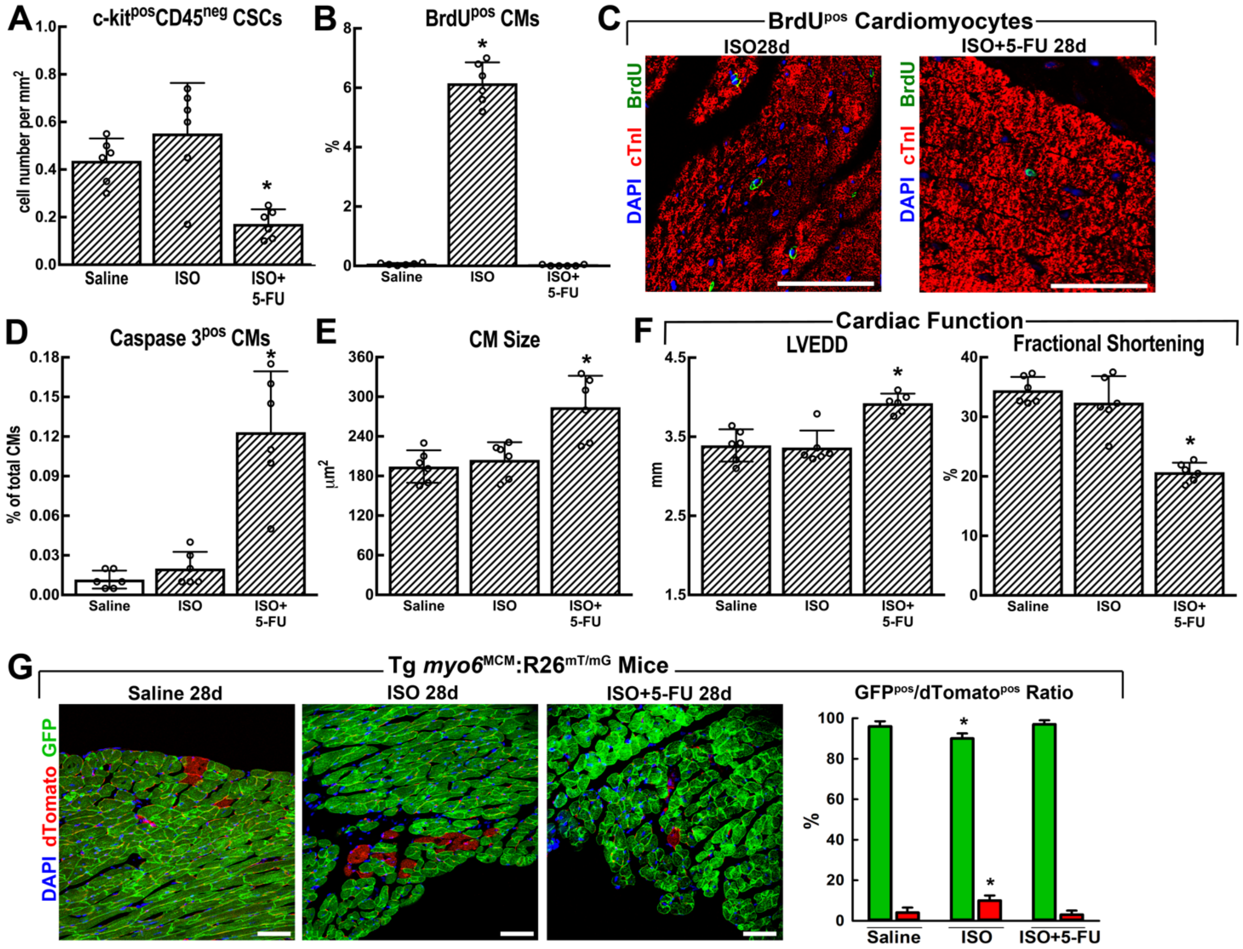

Suppl. Fig. 5. Cardiac Progenitor Activation and Ensuing Cardiomyocyte Formation after Isoproterenol Damage is Essential for Cardiac Recovery. A, 5-FU treatment upon ISO injury ablates endogenous CSCs as assessed by quantitative immunohistochemistry of c-kit^{pos}CD45^{neg} resident CSCs in wt C57BL/6J mice 28 days after Saline (*n*=6), ISO (*n*=6) or ISO+5-FU treatments (*n*=6). p<0.05 vs. all (One-way ANOVA analysis with Tukey's multiple comparison test) . **B,** 5-FU treatment upon ISO injury ablates new cardiomyocyte formation as assessed by quantitative immunohistochemistry of BrdU^{pos} CMs from wt C57BL/6J mice 28 days after Saline (*n*=6), ISO (*n*=6) or ISO+5-FU treatments (*n*=6). p<0.05 vs. all (One-way ANOVA analysis with Tukey's multiple comparison test). **C,** Representative confocal microscopy images showing BrdU^{pos} cardiac cells in the apical sub-endocardium of wt C57BL/6J mice 28 days ISO (left) or ISO+5-FU (right). Note the presence of a BrdU^{pos} cardiomyocytes in ISO that are absent in ISO+5-FU. **D,** 5-FU treatment upon ISO injury increases cardiomyocyte apoptosis as assessed by quantitative immunohistochemistry of Caspase-3^{pos} CMs from wt C57BL/6J mice 28 days after Saline (*n*=6), ISO (*n*=6) or ISO+5-FU treatments (*n*=6). p<0.05 vs. all (One-way ANOVA analysis with Tukey's multiple comparison test). **E,** 5-FU treatment upon ISO injury increases cardiomyocyte hypertrophy in wt C57BL/6J mice 28 days after Saline (*n*=6), ISO (*n*=6) or ISO+5-FU treatments (*n*=6). p<0.05 vs. all (One-way ANOVA analysis with Tukey's multiple comparison test). **F,** 5-FU treatment upon ISO injury causes cardiac dysfunction as assessed by Echocardiography in wt C57BL/6J mice 28 days after Saline (*n*=6), ISO (*n*=6) or ISO+5-FU treatments (*n*=6). p<0.05 vs. all. (One-way ANOVA analysis with Tukey's multiple comparison test). **G,** Representative confocal microscopy images of GFP and dTomato fluorescence in the sub-endocardial, apical layer of Tg-myh6^{MCM}:R26^{mT-mG} mice 28 days after Saline (*n*=5), ISO (*n*=6) or ISO+5-FU (*n*=6). Bar=50 µm. Right, fraction of GFP^{pos} and dTomato^{pos} CMs in Tg-myh6^{MCM}:R26^{mT-mG} mice 28 days after Saline (*n*=5), ISO (*n*=6) or ISO+5-FU (*n*=6) *p<0.05 vs. all (One-way ANOVA analysis with Tukey's multiple comparison test). Note[1]: Scale bar 50µm; Note[2]: All data are Mean ± SD.

Expanded Test for Suppl. Fig. 5G:

The lack of CSC-derived CM regeneration in ISO+5-FU wt animals was confirmed using double transgenic Tg-myh6^{MCM}:R26^{mT-mG} mice after TAM treatment as described. CM replacement by non-CM cells

indicated by the GFP$^+$/dTomato$^+$ CM ratio dilution 28 days after ISO (from 97/3 in saline injected to 90/10 after ISO) was practically absent in ISO+5-FU mice where the GFP$^+$/dTomato$^+$ CM ratio (96/4) was no different from to Saline injection (97/3) (Supplementary Figure 5G), as would be expected in the absence of new CM formation from the CSCs. This lack of contribution of new CMs by progenitor cells is associated with chronic myocardial dysfunction after ISO+5-FU injury and confirms the validity of the model to deplete the myocardium of functional CSCs.

SUPPLEMENTARY FIGURE 6

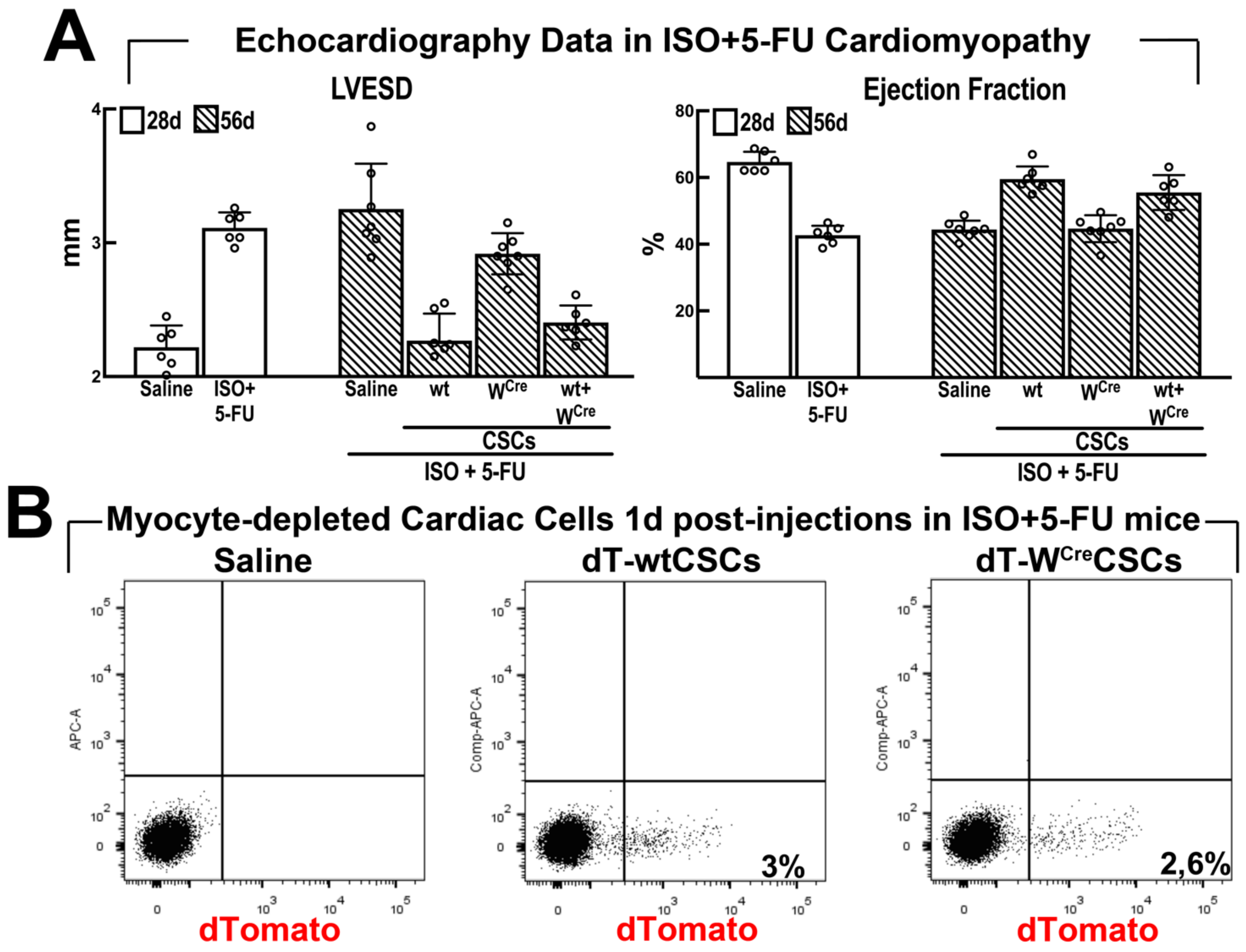

Suppl. Fig. 6. A, Echocardiographic data in male wild type C57BL/6J mice 28 days after Saline or ISO+5-FU (white bars) (*n*=6) and 56 days after ISO+5-FU administration treated with Saline (*n*=7 mice), dT-wtCSCs (*n*=6 mice), dT-W^{Cre}CSCs (*n*=7 mice) and YFP-wtCSCs+dT-W^{Cre}CSCs (*n*=6 mice) injections. *p<0.05 vs. Saline and #p<0.05 vs. ISO+5-FU. (One-way ANOVA analysis with Tukey's multiple comparison test). LVEDD=Left ventricle end diastolic diameter, LVESD=Left ventricle end systolic diameter, EF= ejection fraction. **B,** Flow cytometry analysis of dTomato^{pos} cell engraftment 1 day after Saline, dT-wtCSCs or dT-W^{Cre}CSCs injections in mice with ISO+5-FU cardiomyopathy (*n*=5 mice for each group).

SUPPLEMENTARY FIGURE 7

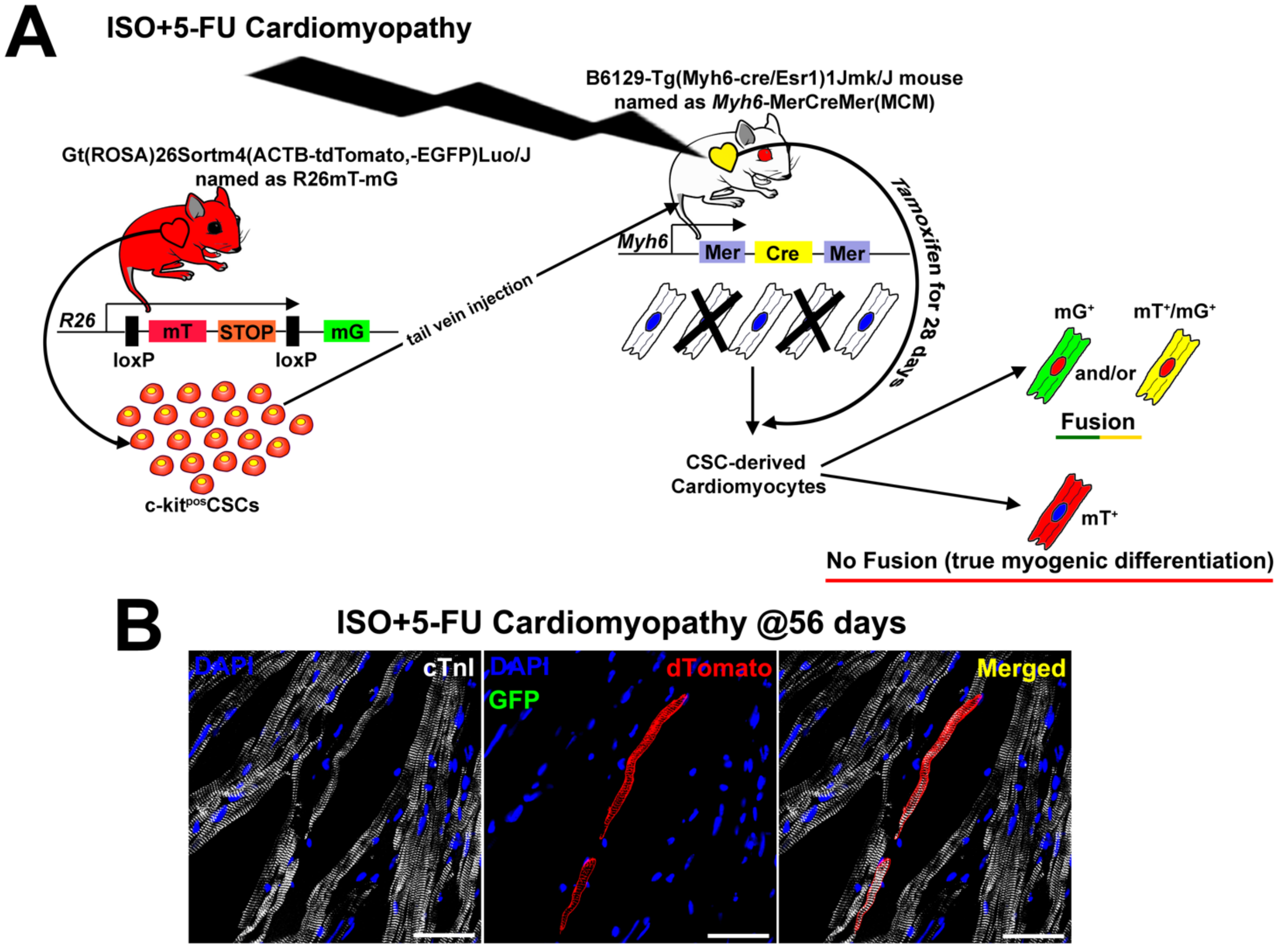

Suppl. Fig. 7. CSCs Differentiate into Cardiomyocytes *in vivo* After Injury with no Evidence of Cell Fusion. A, Schematic of study design (*adapted from reference 18*). Transgenic (Tg) myh6^{MerCreMer} (Tg-myh6^{MCM}) male (*n*=5) mice which carry a transgenic cardiomyocyte-restricted tamoxifen-inducible Cre gene construct, had their myocardium damaged with ISO+5-FU regime. At 28 days after producing the ISO+5-FU cardiomyopathy, mice were treated by tail vein injection with wt clonal dT-CSCs obtained from R26^{mT-mG} male mice. Mice were fed TAM diet for 4 weeks. Thus, these clonal cells and their progeny constitutively express dTomato (mT^{+}) which switches to express membrane GFP (becoming mG^{+}) when recombined in response to Cre Recombinase. The injection of dT-wtCSCs in Tg-myh6^{MCM} mice tests directly whether new myocytes are exclusively the progeny of the wt dT- CSCs injected in which case they should be red (dT^{+}) or the result of cell fusion of the injected cells with host CMs in which case they should be yellow (mT^{+}/mG^{+} = yellow). Indeed, if the putative new CMs were not such but product of the fusion of the injected cells with the host myocytes these fused cells should be yellow or green. TAM-activates Cre only in the host CMs (hCMs) of Tg-myh6^{MCM} and the recombination induces the expression of mG only if the injected reporter-switchable CSCs fuse with the hCMs. Thus, the recombined host CMs will be either mT^{+}/mG^{+} (yellow) or mG^{+} (green), the latter depending on the dilution time of dTomato expression after recombination. On the other hand, mG negative dTomato positive (mT^{+}) CMs can only be the direct progeny of the injected CSCs with NO fusion with host myocytes. **B,** Representative confocal microscopy images of injected CSC-derived newly formed dtTomato^{pos} CMs in mice with ISO+5-FU cardiomyopathy. Note that fluorescently-labelled CMs are only dTomato positive but GFP negative, which excludes cell fusion, instead of *de novo* cardiomyocyte formation from the injected CSCs, as a relevant mechanism for the phenotype of these cells in the ISO+5-FU cardiomyopathy model. Scale bar=50μm.

Supplementary Table 1

Antigen	Antibody ID	Company	Application
c-kit	3C11	Miltenyi Biotec	FC
Rat IgG2b Isopyte control		Miltenyi Biotec	FC
CD45	30F11	Miltenyi Biotec	FC
CD31	390	Miltenyi Biotec	FC
Rat IgG2a Isopyte control		Miltenyi Biotec	FC
BrdU	Bu20a	Biolegend	FC
PCM-1		Atlas Antibodies	FC
Mouse IgG1, K		Biolegend	FC
GATA-4	H113	Santa Cruz	FC
Anti-Rabbit IgG (H+L)		Invitrogen	FC

FC denotes Flow Cytometry

Supplementary Table 2. Echocardiographic data in wild type mice following Isoproterenol injury (see main Figure 2).

ISO 200 mg/kg	*baseline*	*1 day*	*2 days*	*28 days*
**Number (n)**	***15***	***15***	***15***	***13***
**Sex**	male	male	male	male
**Age (weeks)**	12	12	12	16
**Weight (gr)**	27,23 ± 2,17	27,4 ± 2,03	27,66 ± 1,91	29,13 ± 1,55
**HR (bpm)**	488,67 ± 65,96	453,68 ± 28,75	487,78 ± 58,59	490,00 ± 37,71
**LVEDD (mm)**	3,30 ± 0,41	3,15 ± 0,38	3,64 ± 0,29*	3,36 ± 0,28
**LVESD (mm)**	2,17 ± 0,40	2,17 ± 0,26	2,81 ± 0,31*	2,32 ± 0,21
**EF (%)**	64,48 ± 7,95	59,48 ± 8,57	46,32 ± 8,20*	59,2 ± 5,3
**FS (%)**	35,14 ± 6,94	30,92 ± 5,98*	22,77 ± 4,63*	30,74 ± 3,62
**MV E (mm/sec)**	690,40 ± 119,92	709,80 ± 144,15	681,50 ± 130,41	695,54 ± 101,09
**E' (mm/sec)**	-20,17 ± 5,89	-13,69 ± 2,86*	-13,21 ± 3,56*	-19,74 ± 4,71
**E/E' ratio**	-36,18 ± 9	-53,11 ± 12,76*	-53,45 ± 10,16*	-36,74 ± 8,98
**GLS (%)**	-19,60±2,47	-14,61±3,53*	-13,48±3,14*	-18,44±2,86
**GCS (%)**	-21,28+4,09	-19,34±7,77	-18,28±4,20	-19,51±4,32

ISO 400 mg/kg	*baseline*	*1 day*	*2 days*	*28 days*
**Number (n)**	***15***	***15***	***12***	***12***
**Sex**	male	male	male	male
**Age (weeks)**	12	12	12	16
**Weight (gr)**	27,33 ± 1,84	27,41 ±1,9	27,81 ± 2,32	29,2 ± 1,56
**HR (bpm)**	448,67 ± 39,98	454,71 ± 31,05	485,83 ±51,60	481,43 ± 36,71
**LVEDD (mm)**	3,08 ±0,36	3,11 ± 0,50	3,17 ±0,65	3,15 ± 0,35
**LVESD (mm)**	1,99 ±0,26	2,24 ± 0,44*	2,45 ±0,52*	2,10 ± 0,32
**EF (%)**	66,24 ± 4,52	55,52 ±9,81*	45,80 ±8,69*	63,72 ±6,94
**FS (%)**	35,40 ±3,43	28,29 ±6,31*	22,20 ±5,02*	33,90 ± 4,99
**MV E (mm/sec)**	508,53 ± 97,93	599,27 ±157,35	695,13 ± 99,34*	607,17 ± 74,27
**E' (mm/sec)**	-17,49 ± 7,54	-11,32 ±3,26*	-12,51 ±5,07*	-17,07 ± 5,33
**E/E' ratio**	-36,16 ±19,97	-56,3 ±17,86*	-62,27 ±19,52*	-37,73 ± 8,14
**GLS (%)**	-19,90±2,84	-14,06±3,82*	-12,3±2,82*	-18,18±2,27
**GCS (%)**	-24,46±3,08	-22,05±5,69	-17,42±6,44*	-23,22±2,66
